# Chrysin Encapsulated Copper Nanoparticles with Low Dose of Gamma Radiation Elicit Tumor Cell Death Through p38 MAPK/NF-κB Pathways

**DOI:** 10.1007/s12011-023-03596-1

**Published:** 2023-03-11

**Authors:** Lubna O. Abdelhakm, Eman I. Kandil, Somaya Z. Mansour, Sawsan M. El-Sonbaty

**Affiliations:** 1 Biochemistry Department, Faculty of Science, Ain Shams University, Cairo, Egypt; 2 Radiation Biology Department, National Center for Radiation Research and Technology, Egyptian Atomic Energy Authority, Cairo, Egypt; 3 Radiation Microbiology Department, National Center for Radiation Research and Technology, Egyptian Atomic Energy Authority, Cairo, Egypt

**Keywords:** Copper nanoparticles, Low dose radiation, Ehrlich solid tumor, p38 MAPK, NF-κB, CyclinD1, Apoptosis

## Abstract

Improving radiation effect on tumor cells using radiosensitizers is gaining traction for improving chemoradiotherapy. This study aimed to evaluate copper nanoparticles (CuNPs) synthesized using chrysin as radiosensitizer with γ-radiation on biochemical and histopathological approaches in mice bearing Ehrlich solid tumor. CuNPs were characterized with irregular round sharp shape with size range of 21.19–70.79 nm and plasmon absorption at 273 nm. In vitro study on MCF-7 cells detected cytotoxic effect of CuNPs with IC_50_ of 57.2 ± 3.1 μg. In vivo study was performed on mice transplanted with Ehrlich solid tumor (EC). Mice were injected with CuNPs (0.67 mg/kg body weight) and/or exposed to low dose of gamma radiation (0.5 Gy). EC mice exposed to combined treatment of CuNPs and radiation showed a marked reduction in tumor volume, ALT and CAT, creatinine, calcium, and GSH, along with elevation in MDA, caspase-3 in parallel with inhibition of NF-κB, p38 MAPK, and cyclin D_1_ gene expression. Comparing histopathological findings of treatment groups ends that combined treatment was of higher efficacy, showing tumor tissue regression and increase in apoptotic cells. In conclusion, CuNPs with a low dose of gamma radiation showed more powerful ability for tumor suppression via promoting oxidative state, stimulating apoptosis, and inhibiting proliferation pathway through p38MAPK/NF-κB and cyclinD1.

## Introduction

Cancer is the second main reason of death worldwide, which is considered a substantial challenge affecting human communities [1]. Ehrlich solid tumors have been widely used as transplantable models in many investigations due to their contributions to different cancer therapy studies, such as easy examination and investigation of the anticarcinogenic effects of various chemical materials on them [2]. Traditional treatment ways such as chemotherapy have shown various critical and damaging side effects that have adversely affected the patient’s life. In this regard, cancer therapy strategies face many different challenges to offer selective, effective, and safe methods for cancer treatment in order to improve a patient’s life [3].

Radiotherapy (RT) has been utilized in cancer treatment by over 50% of cancer patients during their therapeutic protocol [4]. Radiotherapy involves applying high doses of radiation, 40–60 Gy given as fractionated doses, in order to destroy cancer cells and shrink tumor volume [5]. Radiotherapy use is limited for many problems, including its harmful effect on nearby healthy tissues [6, 7] and radio resistance evolved by many types of malignancies. Some researchers suggested that autophagy could be a potential mechanism for radiosensitization by which cells may protect themselves from ionizing radiation, skipping cell apoptosis, and continuing cell survival, which can remove impaired macromolecules and organelles (e.g., mitochondria) from cells [8]. Chemotherapeutic therapies have been shown to be greater effective when low dose of gamma radiation is used [9]. Low-dose ionizing radiation exposure was found to reduce tumor tissue formation in both experimental animals and humans [10]. In other words, exposure to low dose of gamma radiation is able to inhibit tumor cell progression in Ehrlich tumor-bearing mice [11]via deactivating JAK1/STAT3 pathway. According to several in vitro studies, low dose of ionizing radiation exposure stimulates p53 pathway, cell cycle arrest, apoptosis, immune system enhancement, etc. [12]. Furthermore, improving RT with low doses could be an innovative way to improve RT and other cancer treatment strategies against tumor radio resistance [13]. The dose range of 0.3–0.7 Gy may induce anti-inflammatory effects and secretion of the anti-inflammatory cytokine TGF-β1 [14]. In addition, starting at a dose of 0.5 Gy, proliferation and expression of receptor activator of NF-kB ligand (RANK-L) in fibroblast-like synoviocytes decreased [15].

Radiosensitizers are chemicals or pharmacological substances that can boost tumor cell death by speeding DNA damage and indirectly creating free radicals; at the same time, it is of a lower effect on normal tissues in most cases [16].

Within the last 2 decades, the exponential increase in published papers that have dealt with the use of chemotherapeutic-based nanoparticles (NPs) for cancer treatment indicates the promising prospects of nanotechnology in improved clinical cancer management. Outstandingly, nanotechnology provides a new approach to synthesizing new metal-containing anticancer agents. Recently and because of the medical importance of nanoparticles, they have been synthesized biologically via eco-friendly, cost-effective methods using microorganisms, enzymes, fungus, and plants or plant extracts [17].

Nanoparticles are applied to enhance the effectiveness of radiotherapy for their excellent physical and chemical characteristics, including good biocompatibility, inherent radiosensitization activity, high drug loading, improved tumor tissue permeability, and retention effects [18]. Nano-sensitizers acquired many advantages for radiation dose accumulation and radiation effects enhancement, thereby improving the efficacy of radiotherapy, also increase tumor sensitivity to radiation through reactive oxygen species (ROS) [19]. Among many radiosensitizers, metal-based nano-radiosensitizers have been the most researched. Nanoparticles containing high-Z elements such as Au NPs, Ag NPs, Pt NPs, Bi2S3, Bi2Se3, WS2, and HfO2 have been used as nano-radiosensitizers. High-Z elements have a high radiation absorption ability and may work as radiosensitizers, which absorb more radiation energy than soft tissues and deposit it within tumors throughout the irradiation process, resulting in more damage in tumor cells alone [20]. Worth to mention that radiosensitizers of CuO nanoparticles exhibited the highest DNA damage effect with irradiation [21].

Copper (Cu) is a vital trace element that plays a critical role in growth and replication [22]. However, it is a cofactor for several metalloenzymes as cytochrome c oxidase (COX), which is involved in mitochondrial metabolism, and Cu/Zn-superoxide dismutase (SOD1), which play an important role in cellular radical detoxification against ROS [23]. Many researches detected the elevated demand for copper in tumor cells relative to most other tissues, from that decreasing Cu level was recommended for cancer treatment and protection [24]. Although Cu is cytotoxic, its level is controlled by metallothioneins and glutathione to avoid copper cytotoxicity [25]. Cu compounds are of antitumor activity based on the interactive properties of both copper and the ligand. Copper induces its cytotoxicity through its redox capacities. Cu (I) and Cu (II) redox states’ interconversion in oxidation–reduction cycles. In addition, Cu renders enzyme activity through displacing other ions from the enzyme binding sites. As well, Cu might impair enzyme performance by oxidizing cysteines within iron-sulfur cluster proteins or reacting with hydrogen peroxide to generate ROS in a Fenton-type reaction to produce O2•– or •OH [26]. Cu is of high DNA binding affinity results in promoting DNA breaks [27]. Cu complexes may work as DNA topoisomerase inhibitors of antiproliferative anticancer activity, known as DNA-damaging drugs or poisons that arrest cancer cells in G2/M [28]. Nanomaterials containing copper elements showed marked advantages in many different biomedical, chemical, and physical approaches, so they have been widely used in different fields [29]. Interestingly, copper nanoparticles, which were synthesized using green synthesis methods, have shown various remarkable biological properties and can also be used in different medical approaches [30]. Furthermore, copper nanoparticles can be used as DNA-cleavage agents and potent anticancer therapeutics because of their binding capacity and modifiable surface properties through conjugation with various biomolecules such as proteins and enzymes. Copper nanoparticles can also be used as effective drug delivery nano-formulations and molecular doping systems that act as cancer cell growth controllers [31]. Cu nanoparticles introduced to experimental rats result in oxidative stress and cell death, as detected in increased MDA, superoxide dismutase activity, upregulation of caspases 3, 8, and 9, BCL2-associated X, Bax, and apoptotic peptidase activating factor 1 [32].

In other words, CuNPs may constitute a promising alternative agent for cancer therapeutics in the near future. However, more studies are needed to elucidate the full cellular mechanisms of CuNPs action for the treatment of cancer with higher effectiveness and lower cost.

Natural products are a valuable source of anticancer agents, representing an important alternative remedy for cancer in the twenty-first century. Plant phenolic compounds work as the primary precursors for nanoparticle production and stability through their activity as reducing and stabilizing agents [33]. Chrysin (5,7-dihydroxyflavone) is of flavonoids, naturally present in diverse sources such as honey, propolis, mushrooms, passion fruit, and other plant species [34]. Nonetheless, chrysin shows diverse biological and pharmacological effects, such as anticancer, antioxidant, anti-inflammatory, and antiviral activities. Over and above, chrysin exerts its anticarcinogenic effects through a pleiotropic molecular mechanism(s) of action on cell cycle, inhibits cell proliferation, angiogenesis, and metastasis as well as induces apoptotic processes [35]. Consequently, chrysin could be considered an attractive candidate and/or a starting point for the development of novel anticancer agents.

Shedding the light on the mode of antineoplastic action of CuNPs, the present study aimed to evaluate the activity of copper nanoparticles (CuNPs) synthesized using chrysin as antitumor agent in vitro and as anticancer and radiosensitizer with low dose of gamma radiation in vivo against Ehrlich solid tumor.

## Materials and Methods

### Chemicals

Copper sulfate (CuSO_4_) was supplied by Sigma Chemical Co., St. Louis, USA. Chrysin (5,7-dihydroxyflavone) was obtained from Alfa Aesar Thermo Fisher Scientific Co., Germany. All other chemicals used in the study were of analytical reagent grade.

### Experimental Animals

The current study was performed using eighty Swiss albino female mice weighing 20 ± 5 g, supplied by the Nile Company for Pharmaceuticals and Chemical Industries, animal house breeding unit, Cairo, Egypt. Animals were preserved on a commercial standard pellet diet (21% protein) from Elqaed Company for animal feed and supplied with tap water ad libitum. Maintenance and treatments of animals was authorized by the ethics committee of the National Centre for Radiation Research and Technology.

### Cell Lines

The human breast cancer cell MCF-7 cell line for in vitro study and Ehrlich ascites carcinoma (EAC) cell line for in vivo study were supplied by the Egyptian National Cancer Institute, Cairo University. Ehrlich ascites carcinoma was sustained weekly in Swiss albino female mice via intraperitoneal injection of 0.2 ml containing 2.5 × 10^6^ viable EAC cells diluted by physiological sterile saline solution.

### Tumor Transplantation

Developing a solid tumor was accomplished by injecting Ehrlich ascites carcinoma cells in the right thigh of the mice. The Bright-Line™ Hemocytometer was used for counting Ehrlich ascites carcinoma cells before inoculation. The solid tumor was developed in the right thigh region of the lower limb of each female mouse through intramuscular inoculation of 0.2 ml of EAC cells, containing 2.5 × 10^6^ viable EAC cells.

### Radiation Exposure

Mice whole-body irradiation with gamma radiation was carried out by using ^137^Cesium biological irradiator source, gamma-cell-40 (Atomic Energy of Canada Limited, Chalk River, ON, Canada), at the National Center for Radiation Research and Technology (NCRRT), Egyptian Atomic Energy Authority, Cairo, Egypt. Mice whole-body were irradiated with a single low dose of (0.5 Gy) gamma radiation at 0.653 rad/sec after tumor inoculation.

### Preparation and Characterization of CuNPs

For CuNPs green synthesis, 10 ml of freshly prepared aqueous solution of copper sulfate (1 mM) were reduced by the dropwise addition of chrysin (0.1 mM). The mixture was constantly stirred at 50–60 °C, and an alkaline pH was maintained throughout the experiment. Excess chrysin and CuSO_3_ were removed by dialysis for 24 h. The solution was filtered through a 0.2 μm Millipore filter [36].

Characterization of the prepared CuNPs’ size, shape, and constituents is important for evaluating the biological performance of the newly synthesized nanoparticles. The morphology and size of CuNPs were determined by transmission electron microscopy (TEM), JEOL, model JEM2100, Japan. CuNPs size and size distribution were analyzed by dynamic light scattering (DLS), Malvern Zeta sizer, and Nano ZS ZEN 3600. The chemical structure of CuNPs sample was analyzed by Fourier transform infrared (FTIR) spectroscopy in the range of 400–4000 cm, Equinox 55 IR spectrometer. X-ray diffraction (XRD) analysis was obtained to identify copper incorporation in the synthesized nanoparticle, with an 11 XRD-6000 series, Shimadzu, Japan, using nickel-filter and Cu–Kα target of power 30KW, current 30 mA, voltage 40 kV, and λ 1.5064 Ǻ. CuNPs absorption spectrum was measured by scanning UV–Vis spectrophotometer at a range of 200–600 nm, using ultraviolet–visible spectrophotometer, Jenway UV–Vis spectrophotometer model 6505.

### In Vitro Study

#### CuNPs Cytotoxicity on MCF-7 Cell Line

CuNPs and nano-chrysin’s cytotoxic effects on MCF-7 cells were quantified as 50% cell growth inhibition (IC50) values by MTT assay. MTT (3-(4,5-dimethylthiazol-2-yl)-2,5-diphenyltetrazolium bromide) assay is a colorimetric assay for assessing metabolic activity of MCF-7 cell line as indicator for cellular proliferation, viability, and cytotoxicity in response to CuNPs or nano-chrysin. A 96-well cell culture plate was inoculated with 1 × 10^5^ MCF-7 cells/ml (100 µl/well) and incubated at 37 °C for 24 h to develop a complete monolayer sheet. Media was discarded from the plate then, 50 µl of different concentrations of CuNPs or nano-chrysin (0.5, 1.0, 2.0, 3.9, 7.8, 15.6, 31.25, 62.5, 125, 250, 500, and 1000 µg/ml) and 50 µl of MTT solution were added into each well and incubated for 3 h at 37 °C. MTT solvent (dimethyl sulfoxide) 150 µl was added into each well to dissolve insoluble blue formazan crystals formed by NAD(p)H-dependent oxidoreductase enzyme in viable cells. Colorimetric changes compared to the control without any stimuli were analyzed spectrophotometrically at 590 nm by a microplate reader, Sunrise, TECAN, Inc, USA. A blank cell-free control and an untreated cell control were included in each experiment’s triplicate for each concentration [37].

### In Vivo Study

#### CuNPs Toxicity in Mice (LD50)

An initial necessary step in the assessment of the toxic characteristics of any new compound as CuNPs in vivo is the determination of median lethal dose (LD_50_) value (the dose that kills 50% of the tested animals), which supplies information about the health hazards likely to emerge from short-term drug exposure. Forty Swiss albino female mice were allocated equally in four groups and injected intraperitoneally with increasing doses of CuNPs (0.1, 5, 10, 30, 70, 90, and 100 mg/kg body weight). Mortality was recorded after 24 h. The LD_50_ value was calculated according to [38]. The appropriate dose of CuNPs to be used in vivo was calculated as 1/10 of the LD_50_ value (LD_50_ = 6.67 mg/kg body weight).

### Experimental Design

After 7 days of acclimatization, eighty female mice (20 ± 5 g) were randomly divided into 8 groups (*n* = 10 per group):

1. Control (C) group: Mice were injected intraperitoneally with 0.2 ml of sterile saline.
2. CuNPs group: Mice were injected intraperitoneally with 0.2 ml (0.667 mg/kg b.w.) of CuNPs started from the seventh day of the experiment daily for 2 weeks (5 times each week).
3. Radiation (R) group: Mice’s whole body was irradiated with a single dose of (0.5 Gy) gamma radiation on the eighth day of the experiment.
4. CuNPs/R group: Mice were injected intraperitoneally with 0.2 ml (0.667 mg/kg b.w.) of CuNPs as group 2, and irradiated with a single dose of (0.5 Gy) gamma radiation as group 3.
5. Ehrlich solid carcinoma (EC) group: Mice were inoculated intramuscularly with 0.2 ml of EAC cells (contains 2.5 × 10^6^ viable EAC cells) in the right thigh of the lower limb on the first day.
6. EC/CuNPs group: Mice developed Ehrlich solid tumor and were intraperitoneally with 0.2 ml (0.667 mg/kg b.w.) of CuNPs as group 2.
7. EC/R group: Mice developed Ehrlich solid tumor were irradiated with a single dose of (0.5 Gy) gamma radiation as group 3.
8. EC/CuNPs/R group: EC-bearing mice intraperitoneally with 0.2 ml (0.667 mg/kg b.w.) of CuNPs as group 2, and irradiated with a single dose of (0.5 Gy) gamma radiation as group 3.

**Fig. 1:**
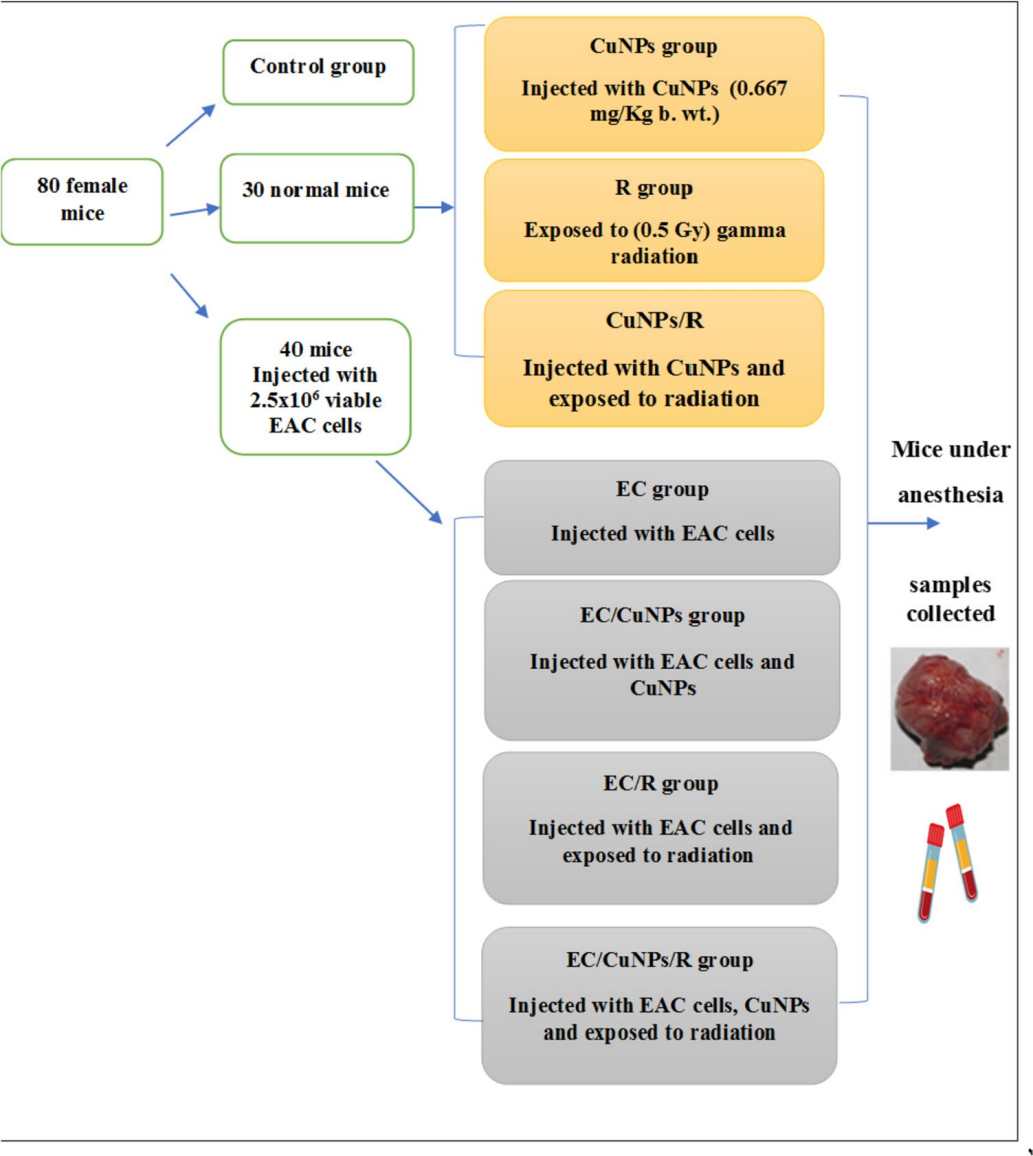
Experimental design and treatment protocol. EC, Ehrlich solid tumor; CuNPs, copper nanoparticles; R, gamma radiation

At the end of the experiment, animals were sacrificed under diethyl ether anesthesia. Blood samples were collected and solid tumors were excised for biochemical and histopathological investigations (Fig. 1).

### Tumor Volume Monitoring

Solid tumor volume was monitored by using Vernier caliper. Tumor volume was measured at the 10th, 15th, and 20th days of EAC cell inoculation and calculated using the equation: tumor volume = 1/2 (length × width^2^), where length represents the greatest longitudinal diameter, while the width represents the greatest transverse diameter [39].

### Biochemical Analysis

#### Kidney and Liver

Serum creatinine was evaluated colorimetrically at 500–550 nm by measuring a colored complex formed of creatinine with picrat in an alkaline medium using a commercial assay kit of Biodiagnostic Company, Egypt.

Alanine aminotransferase (ALT) activity in the serum was measured colorimetrically using the method of the International Federation of Clinical Chemistry and Laboratory Medicine (IFCC). The reaction principle is to convert the pyruvate results from alanine transaminase reaction into lactate by adding lactate dehydrogenase and β-nicotinamide adenine dinucleotide, reduced form (NADH) to the reaction medium. The reaction was monitored at a UV absorbance of 339 nm. A commercial assay kit of QCA, Amposta, Spain, was used.

### Determination of Malondialdehyde (MDA)

Lipid peroxides in terms of malondialdehyde (MDA) were measured in brain tissues according to the method of Yoshioka et al. [40]. The assay depends on the colorimetric reaction of MDA with thiobarbituric acid, giving a pink complex called thiobarbituric acid-reactive substance (TBARS) that can be measured at 532 nm. MDA contents were expressed as nmol/g wet tissue.

### Reduced Glutathione (GSH) Level

GSH level was measured according to Ellman [41]. Protein content of the sample was precipitated by trichloroacetic acid (TCA), and the glutathione in the tumor homogenates was reacted with DTNB (5–5′-dithionitrobenzoic acid; Sigma) to give a yellow chromophore of TNB (thionitrobenzoic acid), spectrophotometrically measured at 412 nm. GSH (Sigma) was used as a standard. GSH concentration was expressed as mg/g wet tissue.

### Catalase (CAT) Activity

CAT activity was assayed in tumor the tissue extracts using colorimetric assay kit of Biotech Inc., Egypt. In brief, samples were diluted in the provided assay buffer and added to the wells of a half-area clear plate. Hydrogen peroxide was added to each well, and the plate was incubated at room temperature for 30 min. The supplied colorimetric detection reagent was added, followed by diluted horseradish peroxidase (HRP), and incubated at room temperature for 15 min. The HRP reacts with the substrate in the presence of hydrogen peroxide to convert the colorless substrate into a pink-colored product. The colored product was read at 560 nm.

### Caspase-3 Level

Caspase-3 level was calorimetrically assayed in the tumor tissue homogenate of each group via ELISA based on sandwich enzyme immunoassay technique using Rat Caspase 3, ELISA kit of Cusabio Co., USA, code no. CSB-E08857r.

### Gene Expression of Nuclear Factor Kappa B (NF-κB), p38 Mitogen-Activated Protein Kinase (p38 MAPK), and Cyclin D_1_

To evaluate the variations of messenger RNA (mRNA) expression of NF-κB, p38 MAPK, and cyclin D_1_ qrt-PCR was performed.

#### RNA Extraction

Total RNA was extracted by using the Qiagen tissue extraction kit, Qiagen, Cat. No./ID:69,504. The concentration of isolated RNA was determined spectrophotometrically by measuring the absorbance at 260 and 280 nm, giving 260/280 ratio > 2.0, using dual-wavelength Beckman, Spectrophotometer, USA. All samples were stored at − 80 °C until the analysis of complementary DNA synthesis reaction.

#### Complementary DNA (cDNA)

Converting total RNA to cDNA was accomplished using the sense and antisense primers, Table 1. Polymerase chain reaction was performed using a high capacity cDNA reverse transcription kit of Thermo Scientific™, Cat. No. FERK1621. Synthesis of cDNA was carried out by adding 3 μl of primers to 10 μl of (1–2 µg) of total RNA and denatured for 5 min at 65 °C in the thermal cycler, then cooled to 4 °C. Then, the reaction mixture was added composed of: 5 μl of first-strand buffer, 2 μl of 10 mM deoxyribonucleotide triphosphates (dNTPs), 1 μl of 40 U/μl RNase inhibitor, 1 μl of 50 U/μl MMLV-reverse transcriptase enzyme, and 10 μl of DEPC-treated water. The mixture was incubated at 37 ºC for 1 h, then at 95 ºC for 10 min, and finally cooled at 4 °C.

**Table 1:** Primer sequence of the studied genes used in qrt-PCR

Gene symbol	Primer sequence (5′–3′)
NF-κB NM_008689.2	Forward: 5′-GAAATTCCTGATCCAGACAAAAAC-3′ Reverse: 5′-ATCACTTCAATGGCCTCTGTGTAG-3′
p38 MAPK NM_001357724.1	Forward: 5′-CGAAATGACCGGCTACGTGG-3′ Reverse: 5′-CACTTCATCGTAGGTCAGGC-3′
Cyclin D_1_ NM_001379248.1	Forward: 5′-TCCGCAAGCATGCACAGA-3′ Reverse: 5′-GGTGGGTTGGAAATGAACTTCA-3′
GAPDH XM_036165840.1	Forward: 5′-ATCACCATCTTCCAGGAGCG-3′ Reverse: 5′-CCTGCTTCACCACCTTCTTG-3′

#### Qrt-PCR Reaction

Was carried out in duplicate in a reaction volume of 25 μl, containing: 2 × SYBR Green PCR Master Mix of Applied Biosystems, Waltham, MA, USA and (2–3 μl) of cDNA; also 900 nM of each primer was added. The thermal cycles conditions applied for each gene were: for NF-κB included preliminary denaturation at 95 °C for 10 min, followed by 36 cycles of denaturation at 98 °C for 30 s, annealing at 60 °C for 60 s, and elongation at 72 °C for 15 s, followed by a final elongation step at 72 °C for 5 min. The thermal conditions for p38MAPK involved 1 denaturation cycle at 95 °C for 2 min, 30 cycles at 95 °C for 1 min, 55 °C for 1 min, and 72 °C for 1 min, and 1 cycle at 72 °C for 5 min. While for cyclin D1 involved initial denaturation at 95 °C for 1 min, followed by 40 cycles of denaturation at 95 °C for 12 s, and annealing at 60 °C for 1 min. Qrt-PCR data analysis was performed by using the Applied Biosystem with software version 3.1, StepOne™, USA. The comparative C_T_ method was used to calculate the relative expression of the target mRNA. To normalize all values, the GAPDH gene was used as the control gene [42].

### Histopathological Examination

Ehrlich tumor-bearing thigh tissue samples preserved in 10% formalin were dehydrated by ethanol and embedded in paraffin wax. For histopathological examination, 5-μm-thick sections were cut and then stained using hematoxylin and eosin [43] before their examination under a light microscope.

### Statistical Analysis

All data were stated as mean ± standard deviation (SD). One-way analysis of variance (ANOVA) was used to analyze the data, followed by the least significant difference (LSD) as a post hoc test. The significance level between mean values was established at *P* ≤ 0.05. SPSS software (version 15.0) was used for all statistical analyses.

## Results

### Characterization of CuNPs

**Fig. 2:**
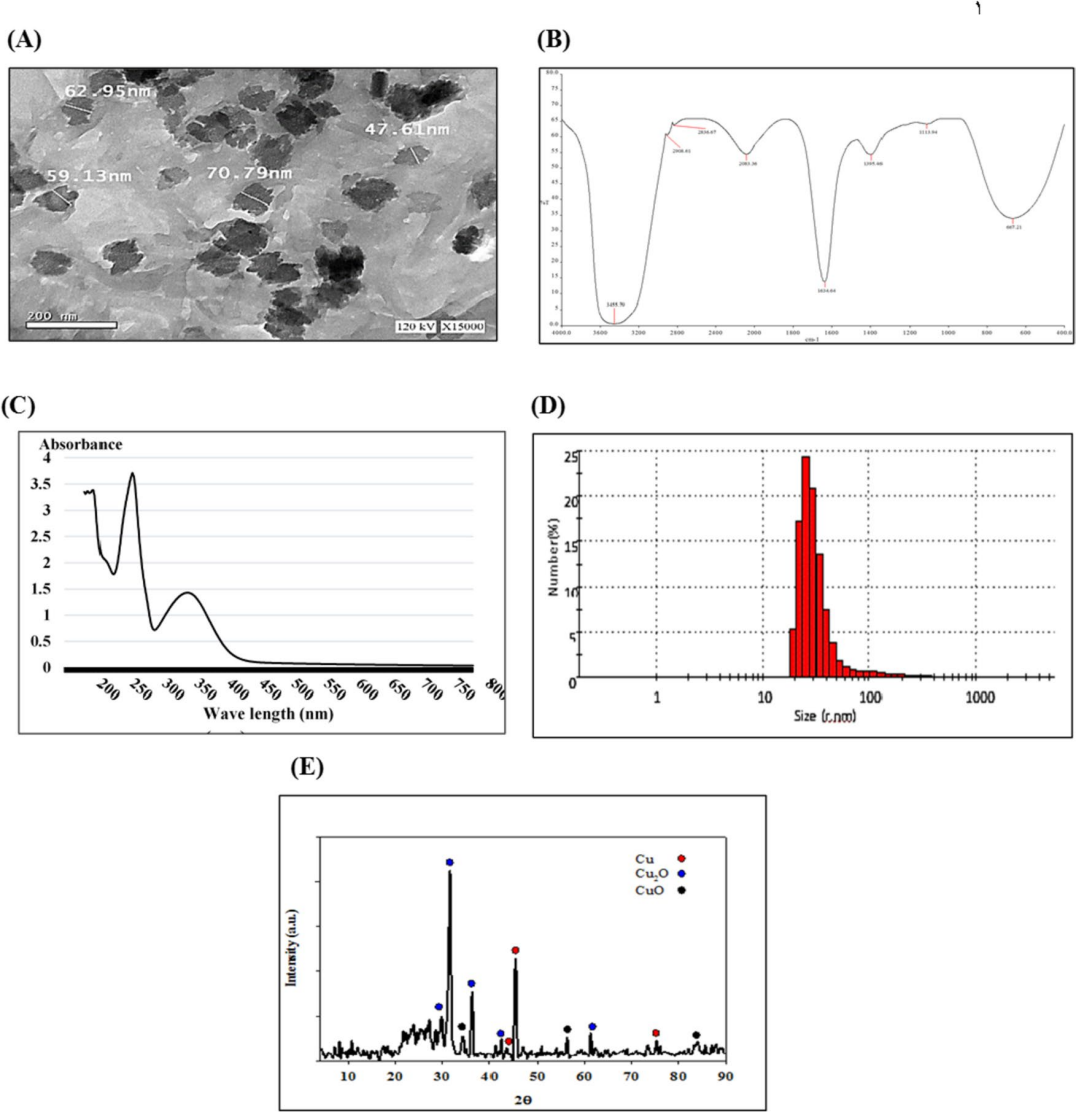
Characterization of synthesized CuNPs through **A** transmission electron microscope micrograph, **B** Fourier transform infrared spectroscopy (FTIR) spectrum, **C** ultraviolet–visible absorption spectrum, **D** dynamic light scattering analysis, and **E** X-ray diffraction diffractogram of CuNPs

Chrysin conjugation to Cu and formation of nanoparticles were analyzed. TEM analysis confirms the size and morphology of synthesized CuNPs. TEM micrographs exhibited an irregular round shape with different sizes ranging from 21.19 to 70.79 nm of CuNPs. FTIR study was performed to ensure the presence of chrysin as coating layer on NPs acting as a capping agent. The FTIR spectrum resented in Fig. 2B revealed strong and medium peaks as follows: a strong broad signal at 3455.79 cm^−1^ (represents hydroxyl group), medium signals at 2908.61 and 2836.67 cm^−1^ (representing C–H bond), a signal at 2083.36 cm^−1^ (represents C–H bending), a strong signal at 1634.64 cm^−1^ (represents C = O bond), a medium signal at 1395.46 cm^−1^ (represents O–H bond), a strong signal at 1113.94 cm^−1^ (represents C–O bond), and a strong signal at 667.21 cm^−1^ (represents C = C bond) indicating the interactions of Cu with chrysin which stabilizing the nanoparticles formed through the surface-bound forming coating layer.

UV–Vis absorbance of CuNPs showed a characteristic surface plasmon absorption band at 273 nm, which indicated the formation of CuNPs, Fig. 2C. DLS analysis revealed CuNPs particle size range from 18.92 to 412.5 nm, with the highest distribution of size 25.37 nm representing 24.3%, followed by the size of 29.39 nm representing 20.8% of the total nanoparticle amount. The polydispersity index (PDI) explains the average uniformity of the particle solution, which was 0.448, indicating acceptable particle size distribution.

Furthermore, the confirmation of CuNPs was made by XRD analysis. The intense and sharp peaks of XRD showed that CuNPs was crystalline in nature, Fig. 2E. The displayed XRD peaks of Cu, CuO, and Cu_2_O were identified according to the International Center for Diffraction Data (ICDD) 01–085-1326, 00–041-0254, and 01–078-2076, respectively. The sample of CuNPs displayed three intensity peaks located at 2*θ* = 43.34°, 45.28°, and 74.99°, corresponding to (111), (200), and (220) face-centered cubic of pure Cu. The crystallized size of Cu was estimated to be 15.73 nm by using Scherrer equation *D* = *Kλ*/(*β* cos *θ*). The five XRD peaks located at 2*θ* = 31.19°, 36.37°, 42.32°, 61.21°, and 73.29° corresponded to the lattice patterns of (110), (111), (200), (220), and (311) for Cu_2_O of crystalized size of 20.65 nm. In addition, the corresponding XRD peaks of CuO located at 2*θ* = at 32.47°, 35.06°, 38.88°, and 83.96° have crystalized size of 21.79 nm.

### In Vitro Study Results

#### CuNPs and Nano-Chrysin Cytotoxicity on MCF-7 Cell Line

**Fig. 3:**
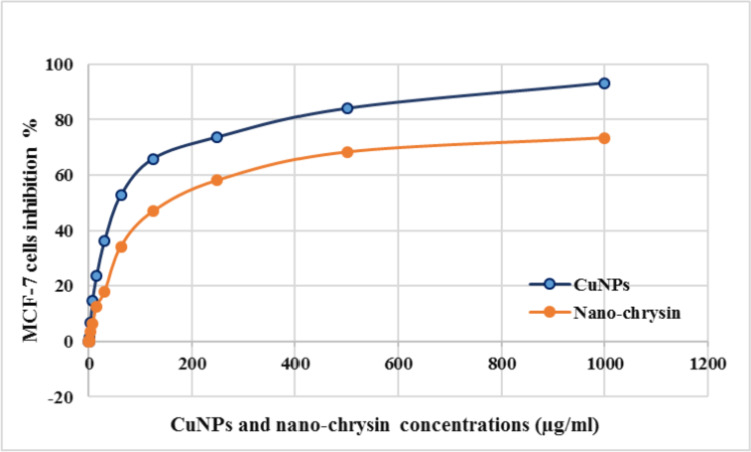
Dose–response curve of MCF-7 cell line when treated with increasing concentrations (0.5–1000 µg/ml) of CuNPs or nano-chrysin. Results plotted as cell death (%) against each CuNPs or nano-chrysin concentrations

The anticancer and anti-proliferative activity of CuNPs was detected in vitro in human breast cancer MCF-7 cell line using MTT assay, Fig. 3. The antitumor activity was expressed as the reduction of cell viability in response to cell exposure to different concentrations of the nanomaterial compared to the untreated control cells. The results exhibited a significant reduction in the proliferation activity of MCF-7 cells treated with CuNPs, recorded IC_50_ value of 57.2 ± 3.1 μg/ml, while nano-chrysin IC 50 was 125.4 ± 6.2 μg/ml (IC_50_ is the concentration that reduced the initial count of viable MCF-7 cell line by 50% compared to untreated control cells).

### In Vivo Study Results

#### Tumor Volume Monitoring

**Fig. 4:**
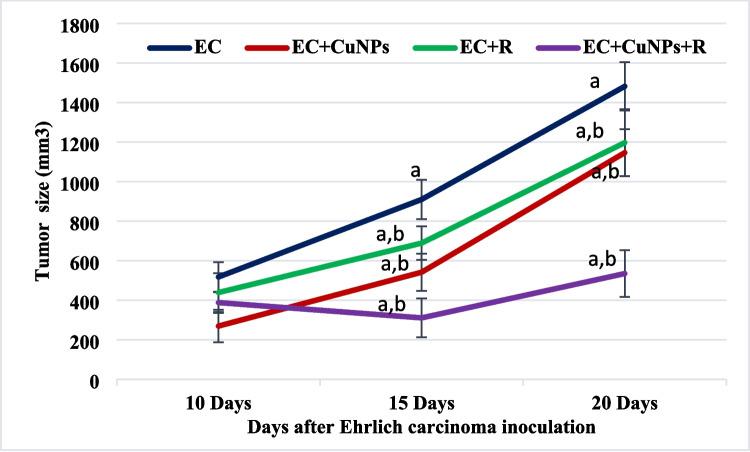
Inhibitory effect CuNPs and low dose γ-radiation exposure on Ehrlich solid tumor size of experimental groups. EC, Ehrlich solid tumor group; EC/CuNPs, Ehrlich solid tumor mice treated with CuNPs group; EC/R, Ehrlich solid tumor mice exposed to 0.5 Gy of whole-body gamma irradiation group; EC/CuNPs/R, Ehrlich solid tumor mice treated with CuNPs and exposed to 0.5 Gy of whole body gamma irradiation group. Data presented as mean ± SD. Data were considered significant at *p* ≤ 0.05. a significant compared to the control; b significant compared to EC group at the same time

Antitumor activity of CuNPs was detected in vivo through monitoring tumor size changes of Ehrlich solid tumor during the time of the experiment, Fig. 4. Ehrlich solid tumor was induced by inoculation of 2.5 × 10^6^ of EAC cells in the right thigh region of the lower limb of female mice. Tumor size of the untreated tumor group (EC group) on the 10th day of inoculation recorded 518.05 ± 274.3 mm^3^, which significantly (*p* ≤ 0.05) increased and reached 1482.2 ± 422.7 mm^3^ on the 20th day of inoculation compared to that of 10th day. A significant (*p* ≤ 0.05) reduction in the tumor size was detected on the 20th day of the inoculation in response to CuNPs and/or low dose gamma radiation exposure (EC/CuNPs, EC/R, and EC/CuNPs/R groups), with percentage of reduction of − 22.6, − 19.21, and − 63.88%, respectively, compared to untreated EC group.

### Biochemical Analysis

**Fig. 5:**
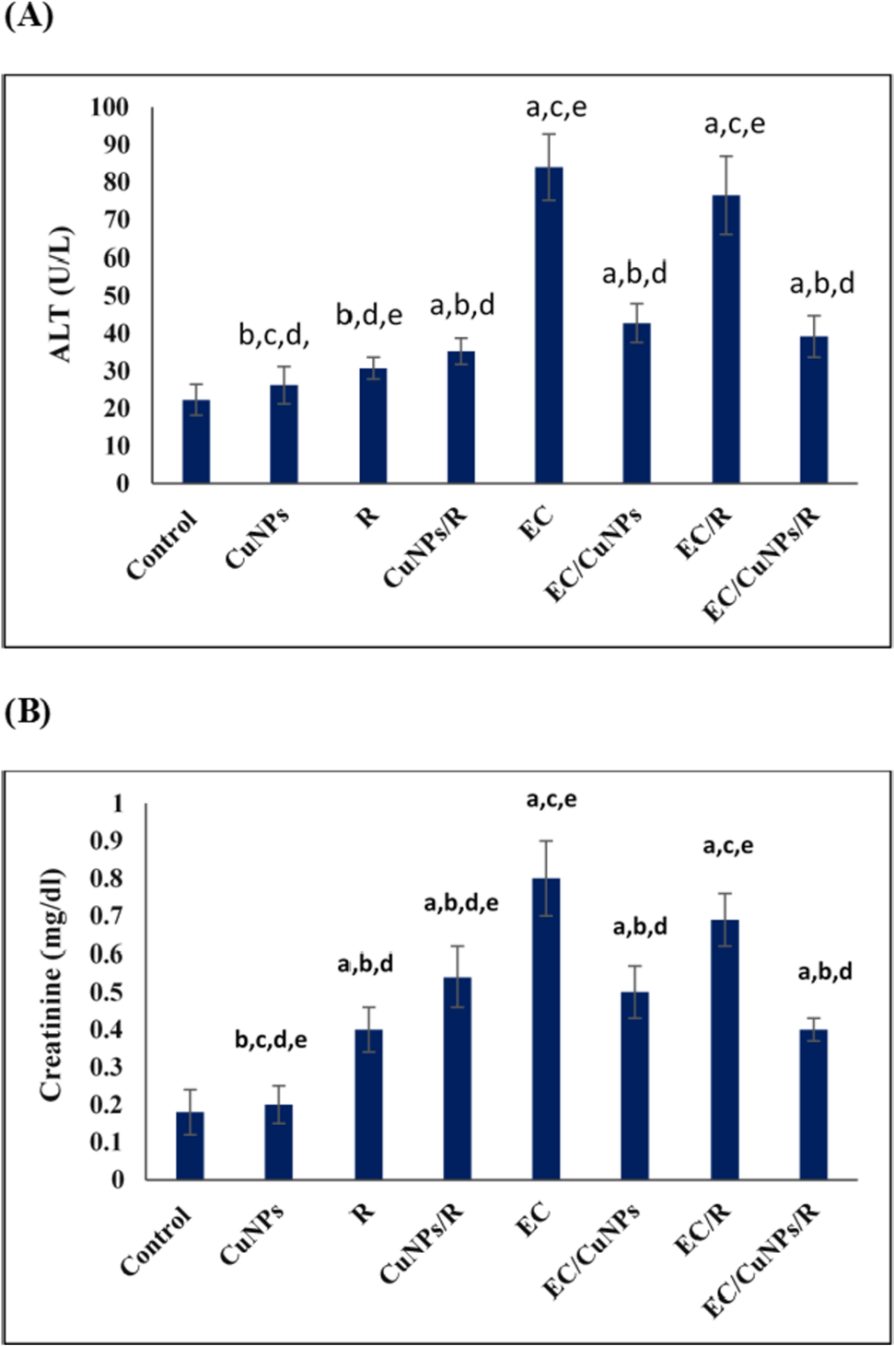
Effect of CuNPs and low dose gamma radiation on **A** ALT activity and **B** creatinine level. Exposed to CuNPs: mice treated with CuNPs, R: mice exposed to whole body gamma irradiation, CuNPs/R: mice treated with CuNPs and exposed to whole body gamma irradiation, EC: Ehrlich solid tumor group, EC/CuNPs: Ehrlich solid tumor mice treated with CuNPs group, EC/R: Ehrlich solid tumor mice exposed to whole body gamma irradiation group, EC/CuNPs/R: Ehrlich solid tumor mice treated with CuNPs and exposed to 0.5 Gy of whole body gamma irradiation group. Data were represented by mean ± SD (*n* = 6). Data were considered significant at *p* ≤ 0.05. Where a, significance compared to control; b, significance compared to EC; c, significance compared to EC/CuNPs; d, significance compared to EC/R; e, significance compared to EC/CuNPs/R

To assess different treatments for toxicity in the liver and kidney, ALT and creatinine were evaluated. Results revealed significant (*p* ≤ 0.05) elevation in ALT activity in the serum of EC group of mice who developed solid tumors by 276.7% when compared to the normal control group. A significant (*p* ≤ 0.05) reduction in ALT activity was detected in tumor-bearing mice treated with CuNPs or exposed to whole-body gamma irradiation or combined treatment of them by − 49.0, − 8.9, and − 53.3%, respectively, compared to EC group, Fig. 5A.

In addition, data analysis of creatinine results revealed a significant (*p* ≤ 0.05) increase in creatinine level in EC group by 172.2% when compared to the normal control group. Whereas, treatment with CuNPs (EC/CuNPs group) showed nonsignificant results with a percent of change of 2.0%, while irradiation exposure (EC/R group) showed an increase in creatinine level with a percent of change of 20.0%. On the other hand, results of creatinine level of EC/CuNPs/R group revealed significant (*p* ≤ 0.05) decrease with percent of change of − 18.4% in comparison to untreated EC group, Fig. 5B.

**Fig. 6:**
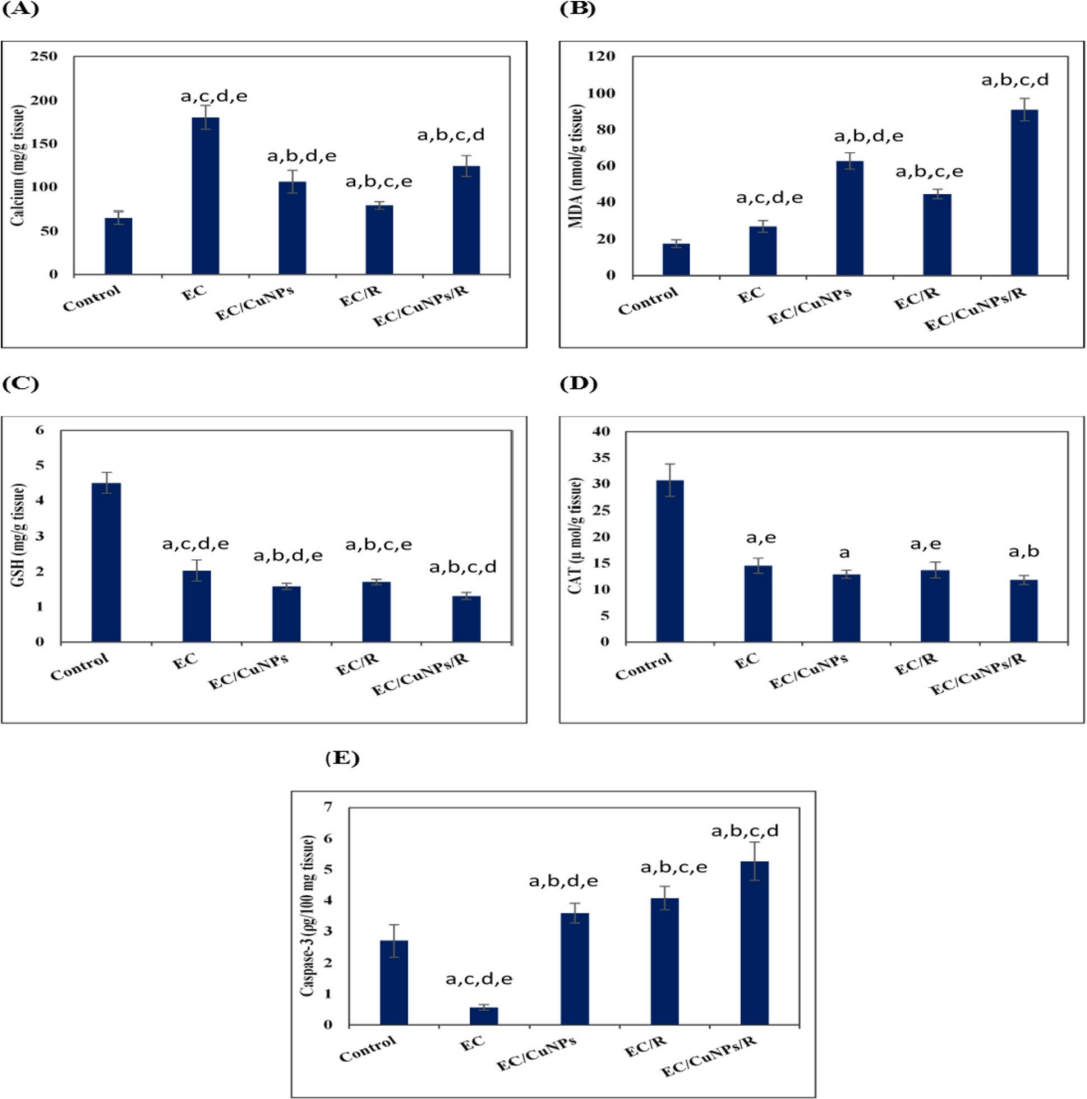
CuNPs and low-dose gamma irradiation exposure effect on mice bearing Ehrlich tumor. Parameters detected in the tumor tissue homogenate were **A** concentration, **B** MDA level, **C** GSH content, **D** CAT activity, and **E** caspase-3. Where EC, Ehrlich carcinoma; CuNPs, copper nanoparticles, and R, low dose gamma radiation. Data were represented by mean ± SD (*n* = 6). Statistically significant differences were assessed by a one-way ANOVA followed by post hoc test. Data were considered significant at *p* ≤ 0.05. Where a, significance compared to control; b, significance compared to EC; c, significance compared to EC/CuNPs; d, significance compared to EC/R; e, significance compared to EC/CuNPs/R

Calcium concentration was detected in the tumor tissue extracts by atomic absorption, Fig. 6A. Our results revealed a significant (*p* ≤ 0.05) elevation in Ca + 2 level in tumor tissue by 176.7% compared to healthy normal tissue. This elevation was significantly (*p* ≤ 0.05) reduced with treatments of CuNPs, irradiation, and the combined treatment (EC/CuNPs, EC/R, and EC/CuNPs/R groups) by − 41.2, − 56.4, − 31.2%, respectively, compared to untreated EC group.

CuNPs and radiation exposure caused changes in oxidative stress status of tumor tissue which was detected by determination of MDA level as a product of lipid peroxidation, Fig. 6B. Results detected a significant (*p* ≤ 0.05) elevation in MDA levels in the tumor tissue by 35.1% compared to that of the control group. Treating tumor-bearing mice with CuNPs, irradiation, and combined treatment (EC/CuNPs, EC/R, and EC/CuNPs/R groups) significantly (*p* ≤ 0.05) elevated MDA levels by 131.7, 64.2, and 236.2%, respectively, compared to untreated EC group.

Tumor cell antioxidant levels are associated with tumor progression and increased resistance to chemotherapeutic drugs. Antioxidant state was estimated by GSH level and CAT activity determination, Fig. 6 C, D. The current data revealed a statistically significant (*p* ≤ 0.05) decrease in GSH and CAT levels in the tumor extract by − 54.9 and − 52.6%, respectively, when compared to the normal tissue. Different treatments of CuNPs and irradiation (EC/CuNPs, EC/R, and EC/CuNPs/R groups) significantly (*p* ≤ 0.05) reduced GSH by − 22.2, − 15.8, and − 35.5%, respectively, while decreased CAT activity by − 11.1, − 5.7, and − 18.5%, respectively, when compared with that of untreated EC group.

The suppression in caspase-3 level in cancer cells pointed to the induction of tumorigenesis. The present data revealed a significant (*p* ≤ 0.05) decrease in caspase-3 level by − 78.9% in the tumor tissue when compared to the control, Fig. 6E. In response to different treatments of CuNPs and irradiation, the level of caspase-3 was significantly (*p* ≤ 0.05) elevated (EC/CuNPs, EC/R, and EC/CuNPs/R groups) by 529.8, 614.0, and 824.6%, respectively, when compared to untreated EC group. Comparing data of treatment groups indicated that combined treatment of greater effect on stimulation apoptosis compared to other treatments.

**Fig. 7:**
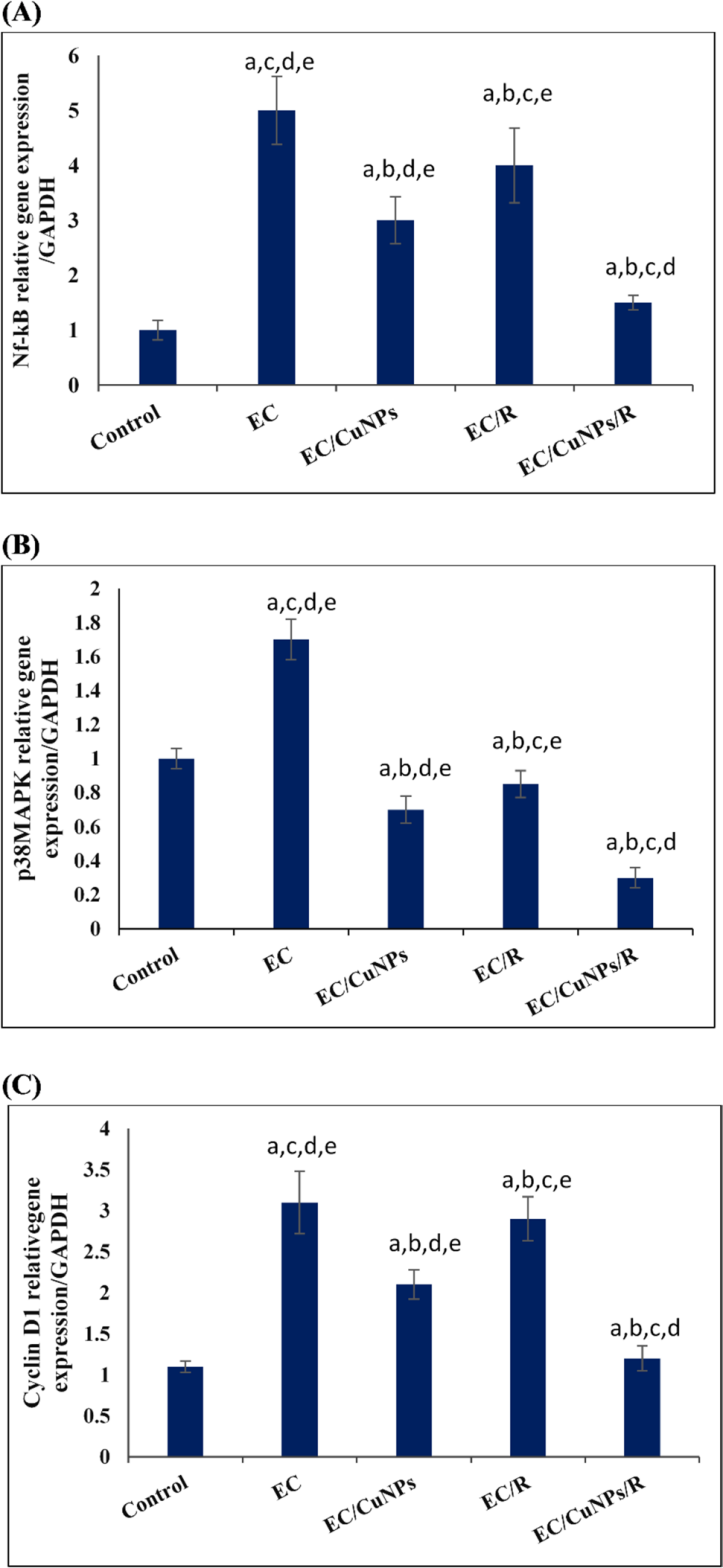
CuNPs and/or low dose γ-radiation exposure downregulated some oncogenes presented in **A** NF-κB, **B** p38 MAPK, and **C** cyclin D1 genes expression in tumor tissues. Where EC, Ehrlich carcinoma; CuNPs, copper nanoparticles; and R, gamma radiation. Data were represented by mean ± SD (*n* = 6). The levels of genes transcription were determined using qrt-PCR. Values are given as mean ± SD (*n* = 6). Statistically significant differences were assessed by a one-way ANOVA followed by post hoc test. Data were considered significant at *p* ≤ 0.05. Where a, significance compared to control; b, significance compared to EC; c, significance compared to EC/CuNPs; d, significance compared to EC/R; e, significance compared to EC/CuNPs/R

Qrt-PCR analysis was performed for detecting the effect of CuNPs and gamma radiation on the gene expression of NF-κB, p38 MAPK, and cyclin D1 in the tumor tissue, Fig. 7 A, B, C, respectively. Results illustrated that gene expression of NF-κB was significantly upregulated by 400 folds compared to the control. On the other hand, comparing different treatments (EC/CuNPs, EC/R, and EC/CuNPs/R groups) to untreated EC group showed a significant reduction in the expression of NF-κB by − 0.4, − 0.2, and − 0.7 folds, respectively, Fig. 7A.

Analyzing Qrt-PCR data pointed to a significant upregulation of p38 MAPK gene expression by 0.7 folds in the tumor tissue of EC group compared to the control. Different treatment groups (EC/CuNPs, EC/R, and EC/CuNPs/R groups) result in a significant downregulation of p38 MAPK gene expression by − 0.6, − 0.5, and − 0.82 folds, respectively, compared to untreated tumor group EC group.

Regarding cyclin D1 gene expression, it showed significant upregulation in the tumor tissue by 1.8 folds compared to the normal control group. A significant downregulation of cyclin D1 was detected in the tumor tissues of different treatment groups (EC/CuNPs, EC/R, and EC/CuNPs/R groups) by − 0.3, − 0.06, and − 0.6 folds, respectively, when compared with untreated tumor group.

Examining the effect of the combination treatment with the single treatment is important. This combination may provide an effect equal (additive), greater (synergistic), or less (antagonistic) than the sum of the individual component effects. The observed value (OV) of the combined extracts and the expected value (EV), which is the mathematical sum of the TAC half values of the individual extracts under test, were compared. If the OV were significantly higher than the EV obtained from the same pair of individual extracts (*p* < 0.05), the combined extract exhibited synergistic interaction. While, if the OV was significantly lower than the EV, the interaction was antagonistic (Habashy et al. 2018) [44].

In view of the results, combined treatment of EC/CuNPs/R was found to be of synergistic effect on MDA and calcium. While it was of additive effect on ALT, creatinine, GSH and CAT, while it was of antagonist effect on tumor volume, Casp-3, Nf-kB, p38MAPK, and cyclin D1, Table 2.

**Table 2:** The observed and expressed values of the combined treatment effects

Effect	Creatinine (mg/dl)		Effect	ALT (U/L)		Effect	Tumor volume (mm^3^)		Combined treatment
	**0.4 ± 0.3**^**a,b**^	**OV**		**39.2 ± 5.6**^**a,b**^	**OV**		**535.3 ± 97.9**^**a,b**^	**OV**	EC/CuNPs/R
**Ad**	**0.43 ± 0.05**	**EV**	**Ad**	**38.98 ± 6.1**	**EV**	**An**	**924.4 ± 106.0**	**EV**	
**Effect**	**GSH** **(mg/g tissue)**		**Effect**	**MDA** **(nM/g tissue)**		**Effect**	**Calcium** **(mg/g tissue)**		
	**1.31 ± 0.08**^**a,b**^	**OV**		**91.1 ± 2.2**^**a,b**^	**OV**		**124.3 ± 12.2**^**a,b**^	**OV**	EC/CuNPs/R
**Ad**	**1.3 ± 0.45**	**EV**	**Sy**	**75.2 ± 7.43**	**EV**	**Sy**	**46.9 ± 2.6**	**EV**	
**Effect**	**NF-κB** **(expression fold)**		**Effect**	**Casp-3** **(pg/100 mg tissue)**		**Effect**	**CAT** **(µM/g tissue)**		
	**0.3 ± 0.03**^**a,b**^	**OV**		**5.77 ± 0.22**^**a,b**^	**OV**		**11.8 ± 0.26**^**a,b**^	**OV**	EC/CuNPs/R
**An**	**0.48 ± 0.5**	**EV**	**An**	**25.59 ± 0.96**	**EV**	**Ad**	**12.2 ± 0.44**	**EV**	
				**P38MAPK** **(expression fold)**			**Cyclin D1** **(expression fold)**		
				**0.3 ± 0.22**^**a,b**^	**OV**		**0.4 ± 0.03**	**OV**	EC/CuNPs/R
			**An**	**0.4 ± 0.03**	**EV**	**An**	**0.63 ± 0.05**	**EV**	

Data are expressed as mean ± SD. *OV*, observed value; *EV*, expected value; *Sy*, synergistic effect; *Ad*, additive effect; *An*, antagonistic effect. Where a, significance compared to control and b, significance compared to EC

## Histopathological Examination

**Fig. 8:**
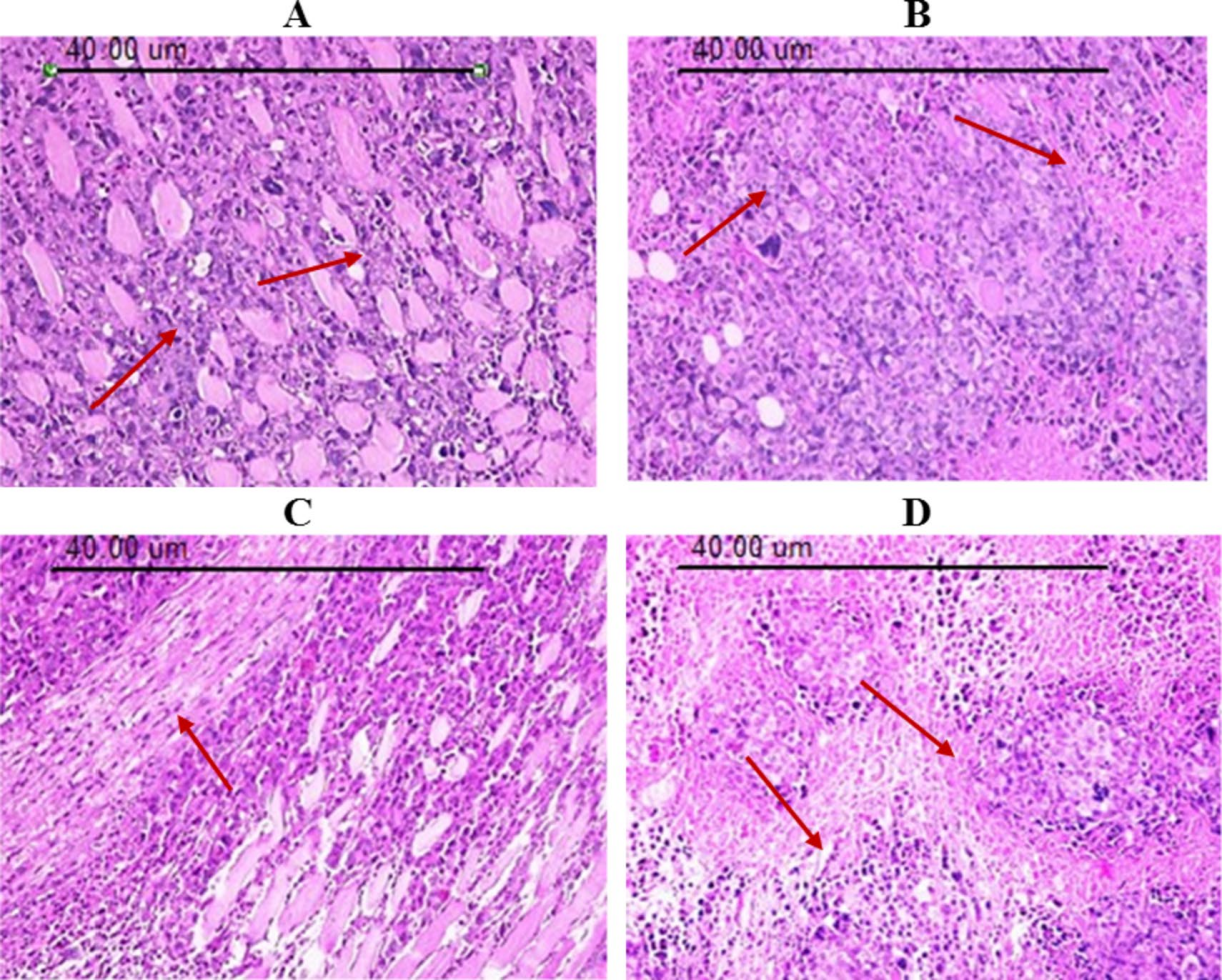
Photomicrographs of Ehrlich solid carcinoma sections stained by hematoxylin and eosin (H&E × 200). **A** EC group, section in the tumor tissue of mice bearing Ehrlich solid tumor. **B** EC/CuNPs group, section in the tumor tissue of EC mice treated with CuNPs. **C** EC/R group, section in the tumor tissue of EC mice exposed to low dose gamma irradiation. **D** EC/CuNPs/R, section in the tumor tissue of EC mice treated with CuNPs and low dose gamma irradiation

Ehrlich solid tumor tissue histopathological examination under the light microscope confirmed the observed inhibition of tumor growth for treated groups in addition to the high growth rate of the control group. The outcomes of EC group revealed compact aggregation of the tumor cells, which spread within the muscular and subcutaneous tissues, arranged in the form of clusters or sheets with deeply basophilic pleomorphic nuclei with nuclear vesicularity. Ehrlich solid tumor showed groups of large, round, and polygonal cells with pleomorphic shapes, hyperchromatic nuclei, binucleation, and anisocytosis. A moderate number of newly formed blood capillaries (neovascularization) were seen in the surrounding tissue. The tumor mass is surrounded by a massive area of necrosis without inflammatory reaction [2], Fig. 8A. EC-bearing mice treated with CuNPs (EC/CuNPs group) showed degenerative changes in the form of vacuolation of neoplastic cells. Pyknosis and karyolysis of Ehrlich tumor cells appeared markedly in the central regions of the tumors. Numerous numbers of inflammatory cells, mainly lymphocytes, macrophages, and multinucleated tumor giant cells, invaded the necrotic areas, and few fibrous tissue proliferation, Fig. 8B. EC-bearing mice treated with low-dose gamma radiation exposure (EC/R group) revealed minimal improvement in comparison with the untreated group. Ehrlich solid tumor cells appeared as a large aggregation of deeply basophilic tumor cells scattered subcutaneously and between the muscular bundles. Some neoplastic cells have pyknotic nuclei with minimal necrotic foci. The inflammatory response appeared in the form of leukocytic infiltration, mainly lymphocytes and macrophages with thick bundles of fibrous tissue, Fig. 8C. EC-bearing mice treated with the combination of CuNPs and low-dose gamma radiation exposure (EC/CuNPs/R group) revealed a high regression of neoplastic mass development and minimal tumor cell infiltrations, extensive necrosis, and apoptosis at the margin of the tumor mass. Marked proliferation of fibrotic tissues and intense leukocyte aggregations, edema, and hemorrhage, Fig. 8D.

## Discussion

Combined chemoradiotherapy has been demonstrated to enhance the efficiency of both chemotherapy and radiotherapy, thus improving cancer treatment. Beyond this, our hope is pinned on tailoring an optimized preclinical therapeutic program based on the biology of the disease and further the evolutionary principles of tumor development. In this connection, such trial was projected to formulate a novel Cu-based nanocomplex under a direct molecular reduction mechanism using the nontoxic and eco-friendly phytochemical chrysin as a strong reducing and stabilizing agent. This biomimetic process completely complied with the principles of green chemistry as it used water as a benign solvent over the course of the preparation and storage of chrysin CuNPs.

Former studies have revealed that copper complexes have the potential to be created as targeted therapies for cancer due to the higher copper uptake by cancer cells compared to normal cells, also its potential function as radiosensitizer, which enhances tumors response to radiation even at relatively low concentrations in the presence and absence of oxygen [6, 45, 46].

Copper nanoparticles in this study were produced via eco-friendly method using chrysin. To establish their fine nanostructure, the biosynthesized CuNPs were characterized via a panel of physicochemical analyses such as TEM, FTIR, UV/VIS spectrophotometer, DLS, and XRD techniques. Analyzing TEM and DLS data size revealed that CuNPs size range results of TEM were 21.19 to 70.79 nm, while DLS analysis detected particles size range from 18.92 to 412.5 nm with size distribution more than 90% for nanoparticles of sizes < 100 nm with homogenous distribution indicated with PDI of 0.448. TEM measures the physical size of the electron dense core, whereas DLS reports the hydrodynamic size of the particle which includes a diffuse layer around the particle indicating bigger sizes.

Nanoparticle size and shape are very important criteria for therapeutic activities. In the present work, CuNPs have elaborated a therapeutic activity regarding the size, as tumor cells were able to accumulate circulating nanoparticles smaller than 150 nm compared to normal cells [47]. Smaller nanoparticles (1–100 nm) can interact with plasma membranes and penetrate and pass through the cell membrane via phagocytosis, endocytosis, or micropinocytosis [48]. Nanoparticle shape plays an important role in cell bioactivity and response, as different shapes induce various cellular responses [49]. Our TEM and XRD results exhibited that CuNPs were of a crystalline form and irregular shape. In other words, nanoparticles are of sharp shape, high surface roughness, and crystalline phase that increase their cytotoxicity when coming into contact with the cell membrane and subsequently induce cell death [50].

CuNPs were characterized with absorption spectral peak at 273 nm, which represents surface plasmon resonance of copper metal nanoparticles [51]. Additionally, the FTIR spectra were examined to determine the surface adsorption of functional groups on nanoparticles [52]**,** and they can also be used to identify possible biomolecules responsible for the capping and stabilization of synthesized metal NPs. The shifting in the hydroxyl (OH) group, (C = C) band corresponding to the single (C–H) or (C–N) bond, C = O of CuNPs spectra when compared to that of chrysin indicates the coordination occurs between Cu and chrysin.

The biological activity of CuNPs against cancer cells was detected in the human breast cancer MCF-7 cell line. Regarding the in vitro toxicity of CuNPs against MCF-7 cells, the result revealed a significant reduction of cell viability relative to control in a dose–response manner. CuNPs biologically synthesized using brown seaweed *Sargassum polycystum* and copper sulfate exhibited antitumor activity against MCF-7 cells with IC_50_ value equal to 61.25 μg/ml [53]. Copper compounds were found to induce MCF-7 cell death through autophagy [54], cell cycle arrest, and inhibition of topoisomerase I [55]. Chrysin as well has been found of anticancer activity on MCF-7 cells in a dose and time-dependent manner detected by MTT assay, which revealed IC_50_ equal 19.5 µM after incubation for 48 h and 9 µM after incubation for 72 [41]. Chrysin has been found to induce its antitumor activity through upregulating apoptotic pathways [56], inhibit angiogenesis [57], metastasis formation [58], and suppress DNA topoisomerases [59], as well as histone deacetylase.

Ehrlich ascites carcinoma (EAC) is a well-established aggressive murine model specific for mice. Ehrlich solid tumor is a rapidly growing carcinoma developed by inoculation of EAC cells intramuscularly, in which cells increase their nuclear materials continuously at the expense of their normal apoptotic rate [61].

In the current study, mice inoculated with EAC cells showed a significant increase in gained tumor size with respect to the control. Many authors attributed the increase in tumor volume to the massive drop in apoptosis, which contrasted with dramatic changes in cell cycle with increased proliferation rate [62]. CuNPs and gamma irradiation administered to EAC mice significantly reduced tumor volume compared to untreated EC mice group. CuNPs and/or gamma irradiation may induce their antitumor effect through cell division delaying impact and apoptosis stimulation. Histological findings clearly demonstrated regression of neoplastic mass as well as extensive number of necrotic and apoptotic cells. Also, chrysin has been found to reduce EAC tumor size through inhibiting VEGF and inflammatory molecules involved in angiogenesis [63], suppressing DNA topoisomerases [64], and histone deacetylase [65], as well as downregulating tumor necrosis factor α (TNF-α) and interleukin 1β (IL-1β) [66].

CuNPs were found to stimulate oxidative stress in tumor tissue homogenates as detected by MDA elevation and reduce antioxidant state as represented by a reduction in GSH and CAT activity compared to untreated tumor tissue, which eventually results in tumor cell death as detected in histopathological analysis that showed degenerative changes in the form of vacuolation of neoplastic cells in the tumor tissue of CuNPs/EC treated mice. It was reported that CuNPs have the ability to increase cell membrane flexibility, enter inside the cell, and interact with different biomolecules, causing DNA degradation and chromatin condensation. CuNPs can also affect the cell cycle by enhancing (G2/M phase) cell cycle arrest [67]. The release of Cu into tumor cells enhances reactive oxygen species (ROS) as well as nitric oxide (NO) production, which results in DNA fragmentation, induces pro-apoptotic protein upregulation such as Bax, and enhances caspase-3, caspase-8, and caspase-9 generation that motivates cancer cells apoptosis [68] even in solid tumor hypoxic condition [69]. Additionally, chrysin had the ability to reduce STAT3 phosphorylation, which inhibits tumor progression and disrupts hypoxia-induced VEGF gene expression. The combination between chrysin with anticancer drugs improved the cytotoxic effects against various cancerous cells without damaging healthy neighboring normal cells [70]. Whole body Irradiation (0.05 GY) was found to increase the levels of DC-related cytokines (IL-2, IL-12, and IFN-γ), promoting biomolecules structural modification [71], triggering anti-tumor immunological responses such as scavenging reactive chemical intermediates, promoting DNA damage repair, reducing inflammation, inducing selective cell apoptosis or senescence, and upregulating both the innate and adaptive arms of the anticancer immune system, allowing the transfer of naive helper T cells to Th1 cells [72–75]. Low dose of gamma radiation has the ability to reduce the occurrence, growth, and spread of cancers [76–78] as well as increase the effectiveness of radiation.

Ehrlich tumor had the ability to increase AFP, ALT, AST, Bcl2, CEA, cholesterol, creatinine, urea, MDA, PCNA, potassium, triglycerides, TNF-α, and NF-kB levels while reducing catalase, GSH, P53, and SOD [2]. It was found that tumor induction damages many internal organs, including the liver and kidney. Worth to mention that cancer promotes liver and kidney damage through extensive regions of necrosis and congestion, as well as mononuclear cell infiltration in the liver and kidney [79]. The results of ALT and creatinine are in harmony with previous research that detected the ameliorative effect of copper complex nanoparticles on ALT activity and creatinine levels in hepatocellular carcinoma [80]. Additionally, chrysin has the ability to reduce elevated ALT and creatinine levels in rats with chronic kidney disease [81]. Moreover, total body exposure to a low dose of γ-radiation has the ability to modulate the elevated serum ALT and creatinine levels induced by Ehrlich solid tumor inoculation [82].

In addition, results exhibited a significant increase in calcium level in the tumor tissue compared to normal tissue, which was significantly decreased with CuNPs and/or γ-radiation exposure compared to untreated tumor tissue. The disruption of Ca^2+^ homeostasis resulting from alteration of Ca^2+^ channels/transporters/pump activities plays a major role in tumor initiation, development, angiogenesis, and metastasis [83]. Calcium ions are essential for cancer cell proliferation and death, serving as major signaling agents and ultimately determining the fate of the cell. When a normal cell transforms into a cancer cell, there is a significant rearrangement of Ca^2+^ pumps, Na/Ca exchangers, and Ca^2+^ channels. This change promotes increased proliferation while impairing a cell’s ability to die [84]. Copper revealed its cytotoxic activity by enhancing endoplasmic reticulum stress and disrupting calcium ions Ca^2+^ homeostasis [85]. Additionally, chrysin exhibited anti-tumor activity against choriocarcinoma cells through modification of mitochondrial membrane potential, disruption of Ca^2+^ levels in cytosol, enhancing generation of reactive oxygen species (ROS), and inducing lipid peroxidation, which affects intracellular Ca^2+^ homeostasis resulting in cancer cell damage [86]. Radiation therapy employs a cell death mechanism triggered by DNA damage, which activates pro-apoptotic Bcl-2 family proteins and evokes enormous Ca^2+^ release from the endoplasmic reticulum, resulting in cell death [87]. Moreover, low doses of ionizing radiation (0.1–0.5 Gy) were found to suppress intracellular (Ca^2+^) influx in the activated rat basophilic leukemia [88].

MDA, a biomarker for oxidative stress, was elevated in tumor tissue compared to normal controls, as it is the end product of lipid peroxidation and its level is related to tumor progression. [89]. At the same time, Ehrlich carcinoma showed depression in the antioxidant state through GSH content and CAT activity inhibition compared to the normal control. CuNPs and/or low-dose gamma radiation treatment increased oxidative stress state as presented by the elevation in MDA levels and decreased antioxidant state as presented by the depression of GSH content and CAT activity compared to the untreated tumor group. Copper, copper complexes, and CuNPs have been found to stimulate oxidative stress and induce ROS generation, resulting in MDA elevation. At the same time, they reduce antioxidant activity by decreasing GSH content and CAT activity [90, 91], enhancing DNA destruction, and stimulating different signals, resulting in apoptosis and cell death [92, 93]. Furthermore, low doses of ionizing radiation induce antioxidant protective mechanisms through modulation of various antioxidant levels such as GSH and SOD, increasing anti-cancer activity, enhancing p53-related apoptosis of destroyed cells and precancerous cells apoptosis through reactive oxygen species (ROS)/reactive nitrogen species (RNS) and cytokine signaling, as well as inhibiting inflammation disease [94]. Additionally, combined treatment of nanocomposite with low dose exposure of gamma radiation was reported to induce cancer cell damage by increasing MDA while decreasing GSH, CAT, and SOD in Ehrlich tumor tissue [95].

Induction of oxidative stress plays an important role in stimulating apoptosis in cancer cells. Ehrlich tumor expressed lower level of caspase-3 compared to normal control, CuNPs, and/or low dose γ-radiation significantly elevated caspase-3 compared to that of untreated tumor tissue. Copper and CuNPs have the ability to stimulate apoptosis by caspase-dependent and independent pathways through induction of pro-apoptotic protein (Bax) and suppression of anti-apoptotic protein (Bcl-2) [66, 90, 96], while chrysin activates apoptosis through intrinsic pathways of apoptosis in view of p53 and Bak, caspase-9 and caspase-3 enhancement, and Bcl2 downregulation [96]. On the other hand, gamma rays may provoke apoptosis through ROS [97].

Ehrlich tumor tissue significantly stimulated oncogenes expression of NF-κB, p38 MAPK, and cyclin D1 compared with normal control. CuNPs and/or gamma radiation modulated gene expression involved in the promotion and growth of cancer cells. Generally, p38 MAPK promotes the downstream induction of transcription factors such as NF-κB, which is essential for cancer cell survival, invasion, and migration, whereas its inhibition promotes activating caspases-3, 9, and 8 in colon cancer cells [98].

Copper has been evaluated as an activator and essential cofactor for MAPK/ERK kinase (MEK), as well as a key molecule in the BRAF/MEK/ERK pathway [99]. Copper antiproliferation activity works by inhibiting the non-canonical and canonical NF-κB pathway through ROS production, preventing IKK kinase complex phosphorylation of the inhibitory units (IκBα) associated with NF-κB [100]. On the other hand, copper nanoparticles coated with phenols or flavonoids such as curcumin alleviated copper toxicity and exhibited antiangiogenic and anti-inflammatory activity through suppressing TNF-α, IGF1, VEGF, IL-6, FGFb, TGFβ, and EGF [101]. While, chrysin showed antiproliferative activity by suppressing p38 MAPK expression, which involved JNK and NF-κB in cardiac cells [102]. Exposure to low dose of ionizing radiation was found to suppress cancer cell proliferation via suppressing p38 kinase in MCF-7 human breast cancer cells [103], inhibiting JNK as well as ERK phosphorylation [87], and down-regulating NF-κB through suppressing TNFα-induced degradation of inhibitor of kappa B (IκB) protein and release of p65 subunit to the nucleus, reducing NF-κB activity [104].

Cyclin D1 analysis in untreated tumor tissue exhibited provoked expression of Cyclin D1 in contrast to normal control; however, this elevation in gene expression was reduced as a result of CuNPs and/or gamma radiation treatments. Cyclin D1 is a marker of cell proliferation as it regulates the cell cycle transition from the G1 phase to the S phase. Our results go along with a former study [105], which stated that CuNPs induce apoptosis, antiproliferative effect, and cell cycle arrest at the G2/M of human extravillous trophoblast cells. In the same line, copper complexes inhibited proliferation and caused cell cycle arrest at G1 phase via down-regulation of cyclin D1 as well as cyclin E1 and up-regulation of cyclin-dependent kinase inhibitors such as (CKIs) p27 protein [106]. Furthermore, chrysin is of antiproliferative activity causes cell cycle arrest on G1 phase and G1/S in skin cancer cell lines in EGF-stimulated JB6 P + cells [107]. Low doses of radiation have been shown to affect normal cellular response, especially proliferation. Low dose of ionizing radiation was found to stimulate cyclin D1 and may induce adaptive resistance [108]. This effect was markedly attenuated by combined treatment with CuNPs. In other words, the combined treatment revealed a stronger inhibitory effect on cyclin D1 expression, causing cell cycle arrest, than each treatment alone.

Histopathological examination of tumor tissue sections illustrated the stimulatory effect of combined treatment on necrosis and apoptosis found all over the tumor tissue. These results are attributed to the ability of CuNPs to promote anti-angiogenic, apoptotic, and necrotic activities [109].

## Conclusion

Copper nanoparticles and copper complexes could be potential chemotherapy against cancer cells due to their high uptake by cancer cells and their potential effect as radiosensitizers even at low doses, in the presence and absence of oxygen (hypoxia) [6, 45, 46]. The use of nanoparticle-based drug delivery system is regarded as a viable strategy for increasing the bioavailability of chrysin and allowing the use of copper and low dose of gamma radiation in cancer therapy through using a newly synthesized CuNPs as a radiosensitizers combined with exposure to low dose of radiation. Based on the results obtained in the present study, the following mechanisms may be behind the anti-tumor effects of the combined treatment of CuNPs and low dose of gamma radiation, as they synergize in inducing oxidative stress state, which triggers signaling pathways of intrinsic apoptosis as well as antitumor and antiproliferative activities via Nf-kB/p38 MAPK signaling, and cell cycle arrest via cyclin D1. Hopefully, our data provides therapeutic rationale for using CuNPs capped by chrysin as adjuvant therapy with low dose of gamma radiation to promote radiotherapy.

## Data Availability

All of the material is owned by the authors, and/or no permissions are required.

## Abbreviations

Cu: Copper
CUNPs: Copper nanoparticles
EC: Ehrlich solid tumor
R: Gamma radiation
ALT: Alanine aminotransaminase
MDA: Malondialdehyde
CAT: Catalase
GSH: Reduced glutathione
RT: Radiotherapy

## Data Availability

The authors confirm that the data supporting the findings of this study are available within the article and/or its supplementary materials.

## References

[1] Bray F, Ferlay J, Soerjomataram I, Siegel RL, Torre LA, Jemal A (2018) Global cancer statistics 2018: GLOBOCAN estimates of incidence and mortality worldwide for 36 cancers in 185 countries. CA Cancer J Clin 68(6):394–424. 10.3322/caac.21492. 10.3322/caac.21492 30207593

[2] Aldubayan MA, Elgharabawy RM, Ahmed AS, Tousson E (2019). Antineoplastic activity and curative role of avenanthramides against the growth of Ehrlich solid tumors in mice. Oxid Med Cell Longev.

[3] Temiz G, Durna Z (2019). Evaluation of quality of life and health care needs in cancer patients receiving chemotherapy. J Cancer Educ.

[4] Begg AC, Stewart FA, Vens C (2011). Strategies to improve radiotherapy with targeted drugs. Nat Rev Cancer.

[5] Hodge JW, Guha C, Neefjes J (2008). Gulley JL (2008) Synergizing radiation therapy and immunotherapy for curing incurable cancers. Oppor Challenges Oncol (Williston Park).

[6] Song G, Cheng L, Chao Y, Yang K, Liu Z (2017). Emerging nano-technology and advanced materials for cancer radiation therapy. Adv Mater.

[7] Choi WH, Cho J (2016). Evolving clinical cancer radiotherapy: concerns regarding normal tissue protection and quality assurance. J Korean Med Sci.

[8] Kuwahara Y, Oikawa T, Ochiai Y, Roudkenar MH, Fukumoto M, Shimura T, Ohtake Y, Ohkubo Y, Mori S, Uchiyama Y, Fukumoto M (2011). Enhancement of autophagy is a potential modality for tumors refractory to radiotherapy. Cell Death Dis.

[9] Zhang Y, Lu Z, Li XY (1999). Effect of combined whole-body low dose irradiation and chemotherapy on growth, metastasis and immune functions in tumor bearing mice. Radiate Prot.

[10] Janiak M, Wincenciak M, Cheda A, Nowosielska E, Calabrese E (2017). Cancer immunotherapy: how low-level ionizing radiation can play a key role. Cancer Immunol Immunother.

[11] Elhadary AA, Marzook EA, Abdelmonem HA (2019). Evaluation of the level of gamma radiation dose on some immune system parameters against cancer. Bioscience J.

[12] Azimian H, Bahreyni-Toossi MT, Rezaei AR, Rafatpanah H, Hamzehloei T, Fardid R (2015). Up-regulation of Bcl-2 expression in cultured human lymphocytes after exposure to low doses of gamma radiation. J Med Phys.

[13] Guozi Y, Wei L, Hongyu J, Xinyue L, Yuguang Z, Dehai Y, Lei Z, Guanjun W, Huimin T, Fujun H, Lu C, Jiuwei C (2016). Low-dose radiation may be a novel approach to enhance the effectiveness of cancer therapeutics. Int J Cancer.

[14] Roedel F, Kley N, Beuscher HU, Hildebrandt G, Keilholz L, Kern P, Voll R, Herrmann M, Sauer R (2002). Anti-inflammatory effect of low-dose X-irradiation and the involvement of a TGF-beta1-induced down-regulation of leukocyte/endothelial cell adhesion. Int J Radiat Biol.

[15] Deloch L, Derer A, Hueber AJ, Herrmann M, Schett GA, Wolfelschneider J, Hahn J, Ruhle PF, Stillkrieg W, Fuchs J, Fietkau R, Frey B, Gaipl US (2018). Low-dose radiotherapy ameliorates advanced arthritis in hTNF-alpha tg mice by particularly positively impacting on bone metabolism. Front Immunol.

[16] Gong L, Zhang Y, Liu C, Zhang M, Han S (2021). Application of radiosensitizers in cancer radiotherapy. Int J Nanomedicine.

[17] El-Sonbaty SM, Moawed FS, Kandil EI, Tamamm AM (2022). Antitumor and antibacterial efficacy of gallium nanoparticles coated by ellagic acid. Dose-Response.

[18] Lim EK, Kim T, Paik S, Haam S, Huh YM, Lee K (2015). Nanomaterials for theranostics: recent advances and future challenges. Chem Rev.

[19] Hernandez-Rivera M, Kumar I, Cho SY, Cheong BY, Pulikkathara MX, Moghaddam SE, Whitmire KH, Wilson LJ (2017). High-performance hybrid bismuth carbon nanotube based contrast agent for X-ray CT imaging. ACS Appl Mater Interfaces.

[20] Choi J, Kim G, Cho SB, Im HJ (2020). Radiosensitizing high-Z metal nanoparticles for enhanced radiotherapy of glioblastoma multiforme. J Nanobiotechnol.

[21] Guerreiro A, Chatterton N, Crabb EM, Golding JP (2019). A comparison of the radiosensitisation ability of 22 different element metal oxide nanoparticles using clinical megavoltage X-rays. Cancer Nano.

[22] Nevitt T, Ohrvik H, Thiele DJ (2012). Charting the travels of copper in eukaryotes from yeast to mammals. Biochim Biophys Acta.

[23] Hordyjewska A, Popiołek Ł, Kocot J (2014). The many “faces” of copper in medicine and treatment. Biometals.

[24] Nair RS, Potti ME, Thankappan R, Chandrika SK, Kurup MR, Srinivas P (2017). Molecular trail for the anticancer behavior of a novel copper carbohydrazone complex in BRCA1 mutated breast cancer. Mol Carcinog.

[25] Culotta V, Scott RA (2016). Metals in cells.

[26] Lloyd DR, Phillips DH (1999). Oxidative DNA damage mediated by copper (II), iron (II) and nickel (II) Fenton reactions: evidence for site-specific mechanisms in the formation of double-strand breaks, 8-hydroxydeoxyguanosine and putative intrastrand cross-links. Mutat Res -Fundam Mol Mech Mutagen.

[27] Marzano C, Pellei M, Tisato F, Santini C (2009). Copper complexes as anticancer agents. Anticancer Agents Med Chem.

[28] Molinaro C, Martoriati A, Pelinski L, Cailliau K (2020). Copper complexes as anticancer agents targeting topoisomerases I and II. Cancers.

[29] Bao YW, Hua XW, Li YH, Jia HR, Wu FG (2018). Hyperthemia-promoted cytosolic and nuclear delivery of copper/carbon quantum dot-crosslinked nanosheets: multimodal imaging-guided photothermal cancer therapy. ACS Appl Mater Interfaces.

[30] Thiruvengadam M, Chung IM, Gomathi T, Ansari MA, Khanna VG, Babu V, Rajakumar G (2019). Synthesis, characterization and pharmacological potential of green synthesized copper nanoparticles. Bioprocess Biosyst Eng.

[31] Halevas EG, Pantazaki AA (2018). Copper nanoparticles as therapeutic anticancer agents. Nanomed Nanotechnol J.

[32] Hu S, Yang J, Rao M, Wang Y, Cheng G, Xia W, Zhu C (2018). Copper nanoparticle-induced uterine injury in female rats. Environ Toxicol.

[33] Amini SM, Akbari A (2019). Metal nanoparticles synthesis through natural phenolic acids. IET Nanobiotechnol.

[34] Naz S, Imran M, Rauf A, Orhan I, Shariati MA, Haq I, Iqra Y, Shahbaz M, Qaisrani T, Shah Z, Plygun S, Heydari M (2019). Chrysin: pharmacological and therapeutic properties. Life Sci.

[35] Saeed S, Jalil TA, Saeideh D (2011). Chrysin reduces proliferation and induces apoptosis in the human prostate cancer cell line pc-3. Clinics.

[36] Li D, Liu Z, Yuan Y, Liu Y, Niu F (2015). Green synthesis of gallic acid-coated silver nanoparticles with high antimicrobial activity and low cytotoxicity to normal cells. Process Biochem.

[37] Wilson JK, Sargent JM, Elgie AW, Hill JG, Taylor CG (1990). A feasibility study of the MTT assay for chemo sensitivity testing in ovarian malignancy. Br J Cancer.

[38] Narang AS, Desai DS, Lu Y, Mahato RI (2009). Anticancer drug development unique aspects of pharmaceutical development. Pharmaceutical perspectives of cancer therapeutics.

[39] Jensen MM, Jørgensen JT, Binderup T, Kjaer A (2008). Tumor volume in subcutaneous mouse xenografts measured by microCT is more accurate and reproducible than determined by 18 F-FDG-microPET or external caliper. BMC Med Imaging.

[40] Yoshioka T, Kawada K, Shimada T, Mori M (1979). Lipid peroxidation in maternal and cord blood and protective mechanism against activated-oxygen toxicity in the blood. Am J Obstet Gynecol.

[41] Ellman GL (1959). Tissue sulfhydryl groups. Arch Biochem Biophys.

[42] Livak KJ, Schmittgen TD (2001). Analysis of relative gene expression data using real-time quantitative PCR and the 2- ΔΔCT method. Methods.

[43] Banchroft JD, Stevens A, Turner DR (1996). Theory and practice of histological techniques.

[44] Habashy NH, Abu Serieb MM, Attiaa WE, Abdelgaleilc SAM (2018). Chemical characterization, antioxidant and anti-inflammatory properties of Greek Thymus vulgaris extracts and their possible synergism with Egyptian Chlorella vulgaris. J Funct Foods.

[45] Kandil E, Aziz NA (2016). Synergistic efficacy of γ-radiation together with gallium trichloride and/or doxorubicin against Ehrlich carcinoma in female mice. Tumor Biol.

[46] Reisz JA, Bansal N, Qian J, Zhao W, Furdu CM (2014). Effects of ionizing radiation on biological molecules mechanisms of damage and emerging methods of detection. Antioxid Redox Signal.

[47] Mozafari MR, Pardakhty A, Azarmi S, Jazayeri JA, Nokhodchi A, Omri A (2009). Role of nanocarrier systems in cancer nanotherapy. J Liposome Res.

[48] Foldbjerg R, Jiang X, Miclăuş T, Chen C, Autrup H, Beer C (2015). Silver nanoparticles wolves in sheep’s clothing?. Toxicol Res.

[49] Jurney P, Agarwal R, Singh V, Choi D, Roy K, Sreenivasan SV, Shi L (2017). Unique size and shape-dependent uptake behaviors of non-spherical nanoparticles by endothelial cells due to a shearing flow. J Control Release.

[50] Sultana S, Djaker N, Boca-Farcau S, Salerno M, Charnaux N, Astilean S, Hlawaty H, De La Chapelle ML (2015). Comparative toxicity evaluation of flower-shaped and spherical gold nanoparticles on human endothelial cells. Nanotechnology.

[51] Raina S, Roy A, Bharadvaja N (2020). Degradation of dyes using biologically synthesized silver and copper nanoparticles. Environ Nanotechnology Monit Manag.

[52] Mudunkotuwa IA, Al Minshid A, Grassian VH (2014). ATR-FTIR spectroscopy as a tool to probe surface adsorption on nanoparticles at the liquid–solid interface in environmentally and biologically relevant media. ANAL.

[53] Ramaswamy SVP, Narendhran S, Sivaraj R (2016). Potentiating effect of ecofriendly synthesis of copper oxide nanoparticles using brown alga: antimicrobial and anticancer activities. Bull Mater Sci.

[54] Koňariková K, Perdikaris GA, Gbelcová H, Andrezálová L, Švéda M, Ruml T, Laubertová L, Režnáková S, Žitňanová I (2016). Autophagy in MCF-7 cancer cells induced by copper complexes. Pharmacol Rep.

[55] Foo JB, Ng LS, Lim JH, Tan PX, Lor YZ, Loo JSE, Low ML, Chan LC, Beh CY, Leong SW, Yazan LS, Tor YS, Howa CW (2019). Induction of cell cycle arrest and apoptosis by copper complex Cu(SBCM)2 towards oestrogen-receptor positive MCF-7 breast cancer cells. RSC Adv.

[56] Li X, Wang JN, Huang JM, Xiong XK, Chen MF, Ong CN, Shen HM, Yang XF (2011). Chrysin promotes tumor necrosis factor (TNF)-related apoptosis-inducing ligand (TRAIL) induced apoptosis in human cancer cell lines. Toxicol In Vitro.

[57] Tsuji PA, Walle T (2007). Benzo[a]pyrene-induced cytochrome P450 1A and DNA binding in cultured trout hepatocytes—inhibition by plant polyphenols. Chem Biol Interact.

[58] Kriengsak L, Hiroaki S, Sherif A, Satoru Y, Takeyuki M, Sirivan A, Amornrat V, Suresh A, Hideo Y, Somsak R, Jisnuson S, Ikuo S (2013). A flavonoid chrysin suppresses hypoxic survival and metastatic growth of mouse breast cancer cells. Oncol Rep.

[59] Russo P, Del Bufalo A, Cesario A (2012). Flavonoids acting on DNA topoisomerases: recent advances and future perspectives in cancer therapy. Curr Med Chem.

[60] Szkudelski T (2001) The mechanism of alloxan and streptozotocin action in B cells of the rat pancreas. Physiol Res 50(6):537–546. https://pubmed.ncbi.nlm.nih.gov/11829314/11829314

[61] Kabel AM, AbdElmaaboud MA (2014). Murine models of cancer. J Cancer Res Treatment.

[62] Bhattacharyya A, Choudhuri T, Pal S, Chattopadhyay S, Datta GK, Sa G, Das T (2003). Apoptogenic effects of black tea on Ehrlich’s ascites carcinoma cell. Carcinog.

[63] Orsolić N, Kosalec I, Basić I (2005). Synergistic antitumor effect of polyphenolic components of water soluble derivative of propolis against Ehrlich ascites tumour. Biol Pharm Bull.

[64] Russo P, Del Bufalo A, Cesario A (2012). Flavonoids acting on DNA topoisomerases: recent advances and future perspectives in cancer therapy. Curr Med Chem.

[65] Szkudelski T (2001). The mechanism of alloxan and streptozotocin action in B cells of the rat pancreas. Physiol Res.

[66] Bai J, Luo Y, Zhanchun S, Fan W, Wang Z, Luan T (2013). Effects and the mechanisms of chrysin on sepsis-associated acute lung injury of rats chrysin inhibits acute lung injury. Life Sci J.

[67] Chakraborty R, Basu T (2017). Metallic copper nanoparticles induce apoptosis in a human skin melanoma A-375 cell line. Nanotechnol.

[68] Dey A, Manna S, Chatt O, Chattopadhyay D, Raj A, Das S, Roy S (2019). Azadirachta indica leaves mediated green synthesized copper oxide nanoparticles induce apoptosis through activation of TNF-α and caspases signaling pathway against cancer cells. J Saudi Chem Soc.

[69] Yun CW, Lee JH, Lee SH (2019). Hypoxia-induced PGC-1α regulates mitochondrial function and tumorigenesis of colorectal cancer cells. Anticancer Res.

[70] Jung J (2016) Emerging utilization of chrysin using nanoscale modification. J Nanomater 2016. 10.1155/2016/2894089.

[71] Song HY, Kim HM, Mushtaq S, Kim WS, Kim YJ, Lim ST, Byun EB (2019). Gamma-irradiated chrysin improves anticancer activity in HT-29 colon cancer cells through mitochondria related pathway. J Med Food.

[72] Feinendegen LE, Pollycove M, Neumann RD (2012). Hormesis by low dose radiation effects: low-dose cancer risk modeling must recognize up-regulation of protection. Springer, Berlin, Heidelberg.

[73] Bobby RS (2014). Radiation-hormesis phenotypes, the related mechanisms and implications for disease prevention and therapy. J cell commun Signal.

[74] Farooque A (2011). Low-dose radiation therapy of cancer: role of immune enhancement. Expert Rev Anticancer Ther.

[75] Shao M, Lu X, Cong W, Xing X, Tan Y, Li Y, Li X, Jin L, Wang X, Dong J, Jin S (2014). Multiple low-dose radiation prevents type 2 diabetes-induced renal damage through attenuation of dyslipidemia and insulin resistance and subsequent renal inflammation and oxidative stress. PLoS One.

[76] Lemon JA, Phan N, Boreham DRJRR (2017). Single CT scan prolongs survival by extending cancer latency in Trp53 heterozygous Mice. Radiat Res.

[77] Sakamoto K (2004). Radiobiological basis for cancer therapy by total or half-body irradiation. Nonlinearity Biol Toxicol Med.

[78] Kaushik N, Kim M, Kim R, Kaushik NK, Seong KM, Nam S, Lee S (2017). Low-dose radiation decreases tumor progression via the inhibition of the JAK1/STAT3 signaling axis in breast cancer cell lines. Sci Rep.

[79] Kapoor R, Gundpatil DB, Somani BL, Saha TK, Bandyopadhyay S, Misra P (2014). Anticancer effect of DL-glyceraldehyde and 2-deoxyglucose in Ehrlich ascites carcinoma bearing mice and their effect on liver, kidney and haematological parameters. Indian J Clin Biochem.

[80] El-Tabl AS, Abd-El Wahed MM, Ashour AM, Abu-Setta MH, Hassanein OH, Saad AA (2019). Metalloorganic copper (II) complex in nano size as a new smart therapeutic bomb for hepatocellular carcinoma. J Chem Cheml Sci.

[81] Ali BH, Al Za’abi M, Adham SA, Yasin J, Nemmar A, Schupp N (2016). Therapeutic effect of chrysin on adenine-induced chronic kidney disease in rats. Cell Physiol Biochem.

[82] Kandil EI, El-sonbaty SM, Moawed FSM, Khedr OMS (2018). Anticancer redox activity of gallium nanoparticles accompanied with low dose of gamma radiation in female mice. Tumor Biol.

[83] Bong AHL (1865). Monteith GR (2018) Calcium signaling and the therapeutic targeting of cancer cells. Biochim Biophys Acta Mol Cell Res.

[84] Stewart T, Yapa KT, Monteith GR (2015). Altered calcium signaling in cancer cells. Biochim Biophys Acta (BBA) Biomembr.

[85] Song YF, Luo Z, Zhang LH, Hogstrand C, Pan Y (2016). Endoplasmic reticulum stress and disturbed calcium homeostasis are involved in copper-induced alteration in hepatic lipid metabolism in yellow catfish Pelteobagrus fulvidraco. Chemosphere.

[86] Park W, Park S, Lim W, Song G (2018). Chrysin disrupts intracellular homeostasis through mitochondria-mediated cell death in human choriocarcinoma cells. Biochem Biophys Res Commun.

[87] Kim YT, Jo SS, Park YJ, Lee MZ, Suh CK (2014). Distinct cellular calcium metabolism in radiation-sensitive RKO human colorectal cancer cells. Korean J Physiol Pharmacol.

[88] Joo HM, Nam SY, Yang KH, Kim CS, Jin YW, Kim JY (2012). The effects of low-dose ionizing radiation in the activated rat basophilic leukemia (RBL-2H3) mast cells. J Biol Chem.

[89] Yu X, Cui L, Zhang Z, Zhao Q, Li S (2013). α-Linolenic acid attenuates doxorubicin-induced cardiotoxicity in rats through suppression of oxidative stress and apoptosis. Acta Biochim Biophys Sin.

[90] Kang Z, Qiao N, Liu G, Chen H, Tang Z, Li Y (2019). Copper-induced apoptosis and autophagy through oxidative stress-mediated mitochondrial dysfunction in male germ cells. Toxicol In Vitro.

[91] Kumar V, Kalita J, Bora HK, Misra UK (2016). Relationship of antioxidant and oxidative stress markers in different organs following copper toxicity in a rat model. Toxicol Appl Pharmacol.

[92] Usman M, Zaki M, Khan RA, Alsalme A, Ahmad M, Tabassum S (2017). Coumarin centered copper (II) complex with appended-imidazole as cancer chemotherapeutic agents against lung cancer: molecular insight via DFT-based vibrational analysis. RSC Adv.

[93] Pramanik A, Pramanik S, Pramanik P (2017). Copper based nanoparticle: a way towards future cancer therapy. Glob J Nanomed.

[94] Scott BR (2014). Radiation-hormesis phenotypes, the related mechanisms and implications for disease prevention and therapy. J Cell Commun Signal.

[95] Abdallah NM, Noaman E, Eltahawy NA, Badawi AM, Kandil E, Mansour NA, Mohamed HE (2016). Anticancer and radiosensitization efficacy of nanocomposite Withania somnifera extract in mice bearing tumor cells. Asian Pac J Cancer Prev.

[96] Santos S, Silva AM, Matos M, Monteiro SM, Álvaro AR (2016). Copper induced apoptosis in Caco-2 and Hep-G2 cells: expression of caspases 3, 8 and 9, AIF and p53. Comp Biochem Physiol C Toxicol Pharmacol.

[97] Zhang Q, Ma S, Liu B, Liu J, Zhu R, Li M (2016). Chrysin induces cell apoptosis via activation of the p53/Bcl-2/caspase-9 pathway in hepatocellular carcinoma cells. Exp Ther Med.

[98] Wang P, Cai Y, Lin D, Jiang Y (2017). Gamma irradiation upregulates B-cell translocation gene 2 to attenuate cell proliferation of lung cancer cells through the JNK and NF-κB pathways. Oncol Res.

[99] Liua H, Zhangb Y, Zhengc S, Wengd Z, Mad J, Lib Y, Xiea X, Zhenga W (2016). Detention of copper by sulfur nanoparticles inhibits the proliferation of A375 malignant melanoma and MCF-7 breast cancer cells. Biochem Biophys Res Commun.

[100] Kenneth N, Hucks G, Kocab A, McCollom AL, Duckett CS (2014). Copper is a potent inhibitor of both the canonical and non-canonical NFκB pathways. Cell Cycle.

[101] Kamble S, Utage B, Mogle P, Kamble R, Hese S, Dawane B, Gacche R (2016). Evaluation of curcumin capped copper nanoparticles as possible inhibitors of human breast cancer cells and angiogenesis: a comparative study with native curcumin. AAPS Pharm Sci Tech.

[102] Mantawy EM, Esmat A, El-Bakly WM, Salah ElDin RA, El-Demerdash E (2017). Mechanistic clues to the protective effect of chrysin against doxorubicin-induced cardiomyopathy: plausible roles of p53 MAPK and AKT pathways. Sci Rep.

[103] Shin J, Woo SH, Lee H, Hong S, Yoo D, Hong S, Lee W, Lee M, Jin Y, An S, Jin D, Park I (2010). Low doses of ionizing radiation suppress doxorubicin-induced senescence-like phenotypes by activation of ERK1/2 and suppression of p38 kinase in MCF7 human breast cancer cells. Int J Oncol.

[104] Li X, Huang Q, Ong CN, Yang X, Shen H (2010). Chrysin sensitizes tumor necrosis factor-α-induced apoptosis in human tumor cells via suppression of nuclear factor-kappaB. Cancer Lett.

[105] Zhang CH, Wang Y, Sun QQ, Xia LL, Hu JJ, Cheng K, Wang X, Fu XX, Gu H (2018). Copper nanoparticles show obvious in vitro and in vivo reproductive toxicity via ERK mediated signaling pathway in female mice. Int J Biol Sci.

[106] Hu K, Zhou G, Zhang Z, Li F, Li J, Liang F (2016). Two hydrazone copper (II) complexes: synthesis, crystal structure, cytotoxicity, and action mechanism. RSC Adv.

[107] Liu H, Liu K, Huang Z, Park CM, Thimmegowda NR, Jang JH, Ryoo IJ, He L, Kim SO, Oi N, Lee KW, Soung NK, Bode AM, Yang Y, Zhou X, Erikson RL, Ahn JS, Hwang J, Kim KE, Dong Z, Kim BY (2013). A chrysin derivative suppresses skin cancer growth by inhibiting cyclin-dependent kinases. J Biol Chem.

[108] Ahmed KM, Fan M, Nantajit D, Cao N, Li JJ (2008). Cyclin D1 in low-dose radiation-induced adaptive resistance. Oncogene.

[109] Mukherjee S, Patra CR (2016). Therapeutic application of anti-angiogenic nanomaterials in cancers. Nanoscale.

